# “*Designer babies*?!” A CRISPR‐based learning module for undergraduates built around the *CCR5* gene

**DOI:** 10.1002/bmb.21395

**Published:** 2020-08-10

**Authors:** Jay N. Pieczynski, Hooi Lynn Kee

**Affiliations:** ^1^ Department of Biology Rollins College Winter Park Florida USA; ^2^ Department of Biology Stetson University DeLand Florida USA

**Keywords:** CCR5, CRISPR‐cas, gene‐editing, germ‐line gene editing

## Abstract

CRISPR‐cas technology is being incorporated into undergraduate biology curriculum through lab experiences to immerse students in modern technology that is rapidly changing the landscape of science, medicine and agriculture. We developed and implemented an educational module that introduces students to CRISPR‐cas technology in a Genetic course and an Advanced Genetics course. Our primary teaching objective was to immerse students in the design, strategy, conceptual modeling, and application of CRISPR‐cas technology using the current research claim of the modification of the *CCR5* gene in twin girls. This also allowed us to engage students in an open conversation about the bioethical implications of heritable germline and non‐heritable somatic genomic editing. We assessed student‐learning outcomes and conclude that this learning module is an effective strategy for teaching undergraduates the fundamentals and application of CRISPR‐cas gene editing technology and can be adapted to other genes and diseases that are currently being treated with CRISPR‐cas technology.

## 1 INTRODUCTION

CRISPR‐cas technology is touted as the “game‐changing technology” for its ability to modify nature at its most fundamental level through the programmable editing of DNA with precision and speed. CRISPR‐cas technology is bound to impact medicine, agriculture, biosphere, and potentially future generations of humans. Broadly speaking, we wondered what the extent of mid to upper‐career science student knowledge was on CRISPR‐cas‐based technology and its uses. Thus, to determine the prior knowledge level of CRISPR‐cas based technologies, we surveyed undergraduate sophomore‐ junior‐ level Genetics students at the onset of the semester. Pre‐surveys indicated that 76.5% of students (13/17) in a Genetics course were minimally aware of CRISPR‐cas technology, while the remainder of the students either had not heard of the technology or were not sure if they had heard of it. Of the students identifying as aware of this technology, our survey results indicated their knowledge concerning CRISPR mostly came from a previous course; however, the news, social media, podcasts/radio and textbooks were also cited as sources for this information. The results of this questionnaire indicated that CRISPR‐cas associated technologies are becoming fundamental components of an undergraduate STEM curriculum, even at the introductory level. Interestingly, although many students had been previously introduced to CRISPR technology, when asked to elaborate on the depth and extent of their knowledge, 66.6% of written responses were coded as “I don't know” or “I can't remember.” Of students remaining, 33.4% of students with a cursory knowledge of CRISPR, comments focused on the novelty and basic components of this technology, however their descriptions lacked accuracy or in‐depth discussion of its applications. Thus, although student knew of its existence or were formally introduced through course work in other courses, this informal survey demonstrated that most students did not have or retain a comprehensive understanding of CRISPR‐cas technology and its diverse uses.

CRISPR‐cas systems, originally identified as an adaptive immune response in bacteria against phages by yoghurt company Danisco,^1^, ^2^ have been repurposed by scientists for genome‐engineering applications in basic science (reviewed in Chen and Doudna.^3^ Recently, the technology provoked a significant reaction that swept through the scientific community. In November of 2018, Dr. He Jiankui claimed to have produced the first human babies born with CRISPR‐cas edited genomes,^4^ affirming predictions that germ‐lined edited “designer babies” were coming soon.^5^ Specifically, germline CRISPR genomic editing technology was purportedly used to create two baby girls, where the gene encoding C‐C chemokine receptor type 5 (CCR5) was manipulated in in vitro fertilized human embryos. CCR5 functions as a G protein‐coupled receptor (GPCR) to chemokines in immune cells, but also acts as a coreceptor for human immunodeficiency virus (HIV).^6^ These “CRISPRed” embryos were implanted, resulting in pregnancy and birth of twin girls with modified *CCR5* genes, and thus have become the first known births of genetically modified humans using CRISPR technology^7^, ^8^ (Table 2). Although Dr. He's work was largely carried out in secret, online documentation showing his study objectives published in the Chinese Clinical Trial Registry^9^ indicates that these types of scientific studies are currently being implemented. The success of these experiments has hastened the debate over the bioethical implications and the guidelines in human heritable germline editing, but also highlighted the immediate need for both current instruction on genome editing technologies and bioethical discussions in the classroom.

Advancements in the ability to edit genomes of organisms have permanently changed the landscape of science education particularly in areas of molecular genetics. In the 1980s, the identification and uses of restriction enzymes and Polymerase Chain Reaction (PCR) ushered in the revolutionary recombinant DNA technology, leading to instructional design and curricula changes in biology education. Thirty plus years later, genomic editing via CRISPR‐cas‐based systems has necessitated further change to curricula. One such example is textbook resources. Many recent editions published just a few years ago lack deep descriptions and applications of CRISPR technologies. Although this is surely to change, the speed of the science will always outpace the rate of textbook publication. From a content perspective, working with recombinant DNA requires students to understand and apply specific fundamental molecular concepts; for example, that DNA is double stranded and antiparallel, the structure and components of plasmids, specificity of restriction enzymes, and the theory of PCR amplification using primers and heat‐stable DNA polymerases. With CRISPR‐cas‐based approaches, students are required not only understand the basic concepts above, but also adequately understand and apply additional molecular concepts such as hybridization between DNA and functional RNA molecules, cas‐nuclease activity, endogenous DNA repair mechanisms, the effect on reading frames/codons, loss‐of‐function and gain‐of‐function effects, and genotype–phenotype relationships.

A bevy of evidence suggests a common set of student misconceptions concerning molecular genetics.^10^, ^11^, ^12^, ^13^, ^14^ Traditionally, instruction has relied upon these core molecular concepts being reinforced in a laboratory setting, where these misconceptions may be addressed through hands‐on application‐based lab work. CRISPR‐cas‐based methodologies have been used successfully to train students in fundamental molecular biology skills^15^ and in a variety of model organisms, including yeast,^16^, ^17^, ^18^ *Escherichia coli*,^19^ *Drosophila melanogaster*,^20^ and *Arabidopsis thaliana*.^21^ However, not all undergraduate teaching laboratories are equipped to sufficiently perform gene editing experiments in live organisms and conduct the subsequent molecular and phenotypic analysis, let alone provide high impact course‐based undergraduate research experiences (CUREs) for students using the technologies. Additionally, not every institution requires genetics or molecular biology courses to have an experiential learning opportunity in the lab. Despite these barriers, evidence suggests that students can achieve meaningful learning gains by performing alternative activities such as interactive computer simulations,^22^, ^23^, ^24^

kinesthetic modeling activities,^25^, ^26^ or case‐based learning modules.^27^

CRISPR‐cas‐based genomic editing tools are becoming commonplace in both research and in higher education, necessitating critical dialogues on ways to effectively bring this technology into the undergraduate classroom. We asked whether we can provide effective instruction on genomic editing technologies in educational settings where wet‐lab resources are unavailable, are cost prohibitive, or in situations that require on‐line learning. Can students still understand and apply the principles of CRISPR without physically performing gene‐editing experiments on organisms or cells in lab? Researchers in lab spend a considerable amount of time devoted to the technical aspects of CRISPR‐cas‐based studies, including designing the tools to generate the type of modification desired, target cells/tissues, germline vs. somatic modification, and mode of delivery of CRISPR‐cas tools. Thus, we sought to build a case‐based learning module that centers around the technical design, implementation, and consequence of CRISPR‐cas gene editing.

**FIGURE 1:**
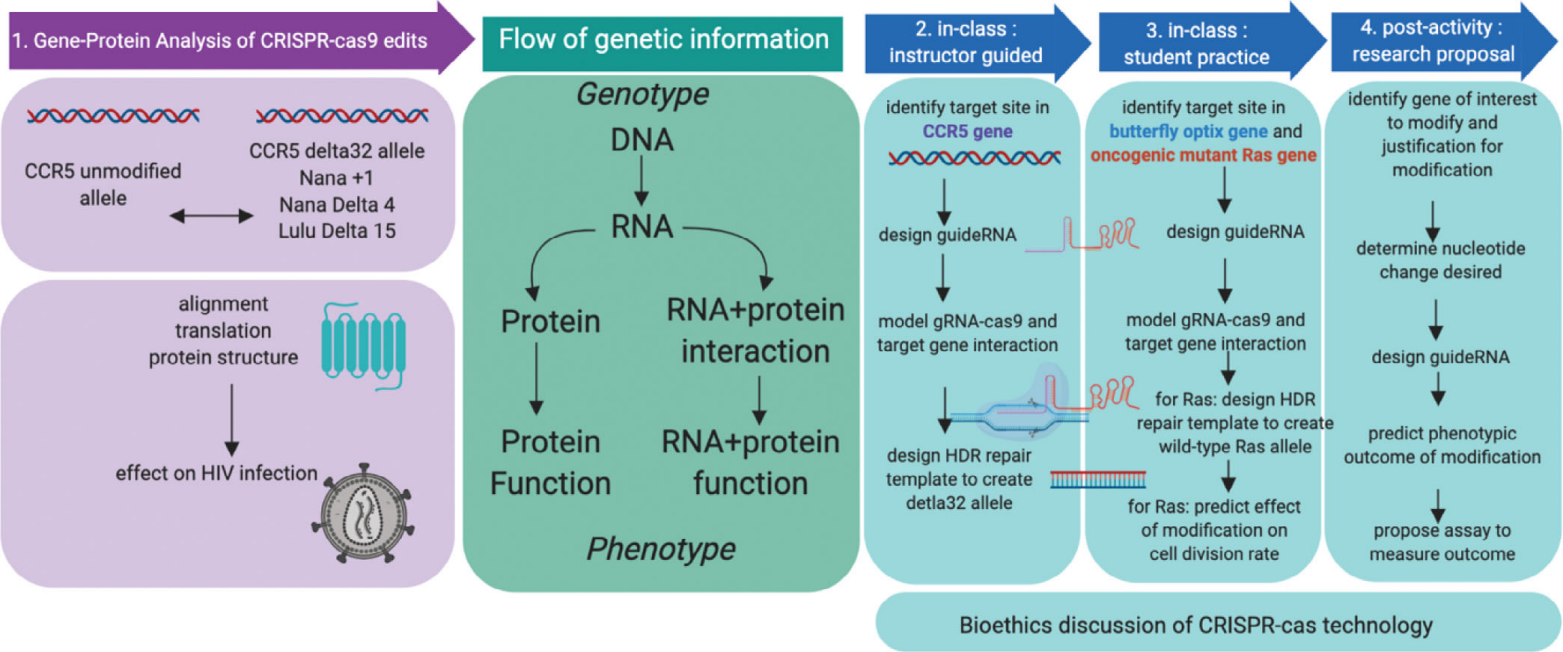
Overview of the CRISPR‐cas case‐based learning module workflow. The center green box shows the flow of the central dogma of molecular biology. The purple boxes on the left show how comparison of unmodified CCR5 allele and modified alleles are compared to enable analysis of the genotypic and phenotypic effects of CRISPR‐cas modifications. The blue boxes on the right show three sequential student activities centered on the technical and application of CRISPR‐cas editing strategy

Using the manipulation of the gene *CCR5* in human embryos as a model example, we describe the design and organization of a gene editing experiential lesson where students apply key concepts in molecular genetics to learn the principles of a specific CRISPR‐cas editing design strategy and analysis of the molecular and phenotypic outcomes, with an intentional focus on the bioethics associated with this technology (Figure 1). We specifically are focusing on the use of the cas9 endonuclease due to its popularity, ease, and ubiquitous use in many CRISPR‐based manipulations. We combined computer‐based work utilizing basic bioinformatics and sequencing software with modeling to educate students on the essential background, planning and design of CRISPR‐cas9‐based edits. We then challenged students to demonstrate their transference of knowledge from the CCR5 case‐based module by having them investigate and propose their own research projects utilizing CRISPR‐cas‐technologies. Our learning outcomes and student work are described in Table 1. When assessed, we determined that our *CCR5* case‐based learning module was an effective method for introducing the fundamental technical aspects of molecular genetics and importantly CRIPSR‐cas system. We found that students are eager to learn about CRISPR‐cas technology and its applications in today's world, and students gain a deeper understanding of the mechanisms behind the technology that has significant power to change human biology.

**TABLE 1:** Learning outcomes and activities for case‐based learning module

	Student learning outcomes students will learn:	Student activities
1	To analyze how different mutations/insertions/deletions due to CRISPR‐cas9 activity affects reading frame, codons, and protein sequence	Align bases of reference sequence to mutant sequences on paper and using DNA computer software (SnapGene) Analyze reading frame, codon and amino acids created Predict and draw proteins created from CRISPR modified alleles
2	To identify appropriate PAM and target sequence for cas9	Identify and label in reference *CCR5* sequence using DNA computer software
3	To design the appropriate gRNA, and understand how guideRNA and cas9 form complex with target sequence utilizing base complementarity	Draw on paper and/or in Biorender(computer) how guideRNA and cas9 form a complex on target DNA strands, and indicate where nuclease activity occurs
4	Difference in molecular outcomes from non‐homologous end joining (NHEJ) and homology directed repair (HDR) after cas9 cleavage	Compare mechanisms of NHEJ and HDR
5	Design an appropriate repair template (for HDR)	Create HDR in SnapGene
6	To apply 1–5 on a new gene	Exercise using Ras Research proposal
7	Discuss the bioethical implications of CRISPR‐cas technology	In‐class discussion

## 2 MATERIAL & METHODS

### 2.1 Intended audience and learning time

This work is intended for sophomore‐ and junior‐level undergraduate Biology students enrolled in a Genetics or Biotechnology course, but can be integrated and modified for Introductory Biology course or upper‐level Molecular Biology course. The main activity was implemented during a 2‐hour 45‐minute laboratory period. The activity can be adapted to fit two traditional 90‐minute class/lecture periods. Subsequent post‐activity student work where students created a research proposal was carried out over the next two laboratory periods and during students' own time.

#### 2.1.1 *Prerequisite student knowledge*

Student should have prior exposure to basic molecular genetics principles that include DNA/RNA structure, codons, reading frames, mutation. However, this lesson can be adapted to introduce and apply the concepts of codons/mutations.

### 2.2 Materials

Each student will require a computer/laptop with access to the internet and the software program, SnapGene Viewer (full version, https://www.snapgene.com). A free 30‐day trial to SnapGene Viewer can be downloaded by each student with their email address. Alternative molecular biology software could be used with modifications to the below resources.

The student handouts and accompanying instructor guide/key are provided in Supporting Information.

### 2.3 Instructions for faculty and students

#### 2.3.1 *Comparative analysis of CCR5 gene and modified allele variants on paper*

The activity utilizes the gene encoding human CCR5; the intended target gene used in Dr. He's study. *CCR5* gene encodes for a chemokine GPCR in T cells, and was targeted because of the known role of the CCR5 as a co‐receptor in HIV infection of humans.^28^ *CCR5* delta 32 (Δ32) is a naturally occurring allele lacking 32 nucleotides that correspond to a sequence that normally codes for part of the co‐receptors second extracellular loop.^28^ Removing these 32 nucleotides results in a premature stop codon due to frameshift, and the resultant truncated protein product can no longer be exocytosed to the cell membrane.^29^ Individuals homozygous for the *CCR5* Δ32 variant are resistant to HIV infection, as CCR5 is required for membrane fusion during HIV infection. The HIV life cycle and the key molecular components of a HIV infection cycle in immune T cell can be introduced to students using this animation by Janet Iwasa.^30^

Dr. He's goal of editing human embryos was to create a similar non‐functional HIV‐resistant CCR5 variants using CRISPR‐cas technology.^31^ However, the results of these experiments have purportedly generated three new *CCR5* allelic variants named after the twin girls, Lulu and Nana, with Lulu being heterozygotic for a novel *CCR5* variant (the Lulu *CCR5* allele) and wild‐type allele, and Nana being heterozygotic at the *CCR5* locus (Nana *CCR5* alleles 1 and 2 respectively).^4^, ^31^ He also claimed that a third child has been genetically modified in a similar manner.

**FIGURE 2:**
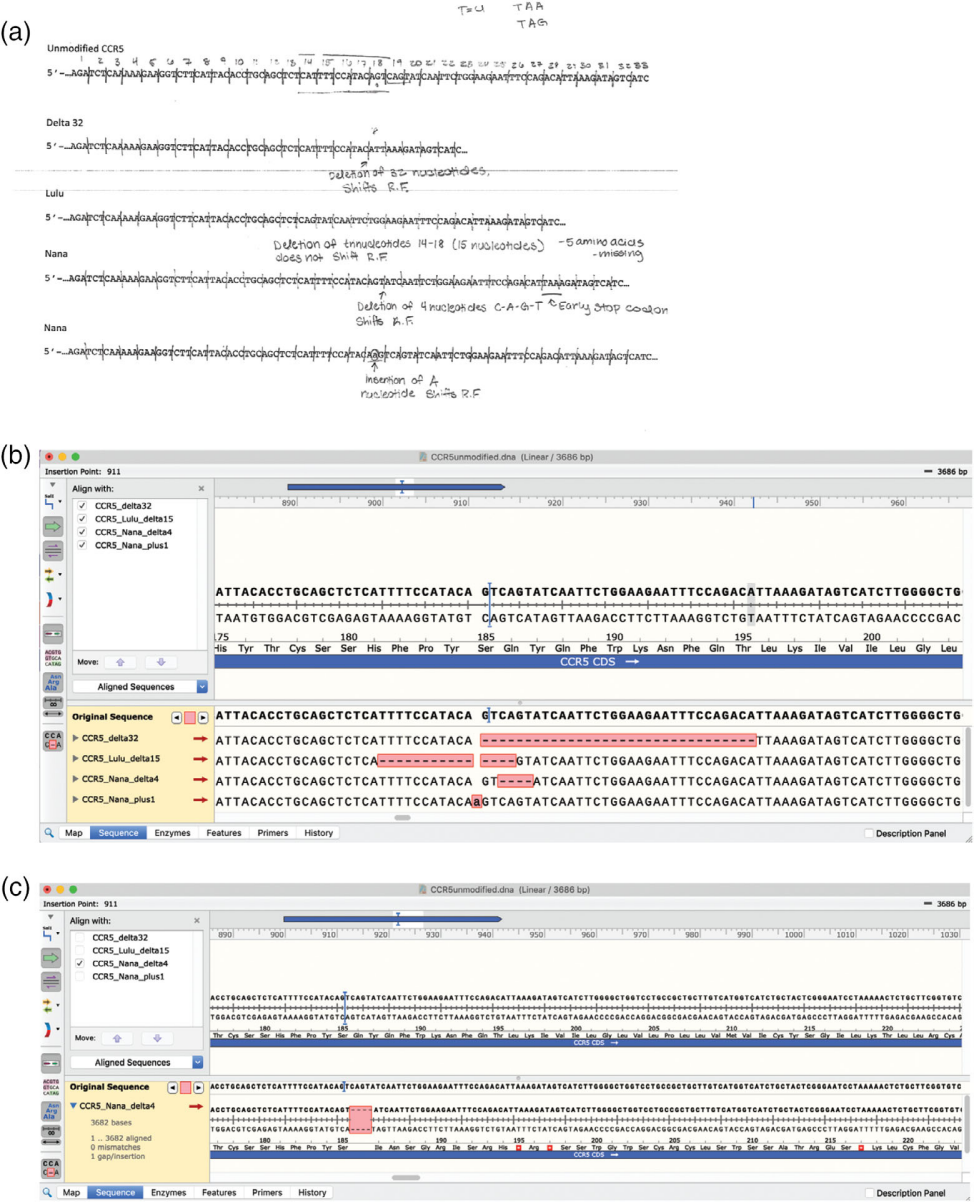
Student work aligning unmodified *CCR5* allele to HIV resistant Δ32 and CRISPR‐cas modified alleles (a) on paper, and (b) using computer software program SnapGene. B) shows all alleles aligned to the unmodified allele. Panel (c) shows an expanded alignment view with the change in reading frame and early stop codon (red asterisks). Expanded alignment views for other modified alleles are shown in Figure S1

To begin their comparative analysis of the different alleles, students were given a worksheet (Supporting Information) that contains the partial coding DNA sequences of an unmodified *CCR5* allele, the HIV resistant allele Δ32 *CCR5*, and three novel alleles generated via CRISPR‐cas9‐based modification. They were tasked to determine the nucleotide differences between them and the effect on the reading frame, codon and amino acid (Figure 2a, example of student work; Table 2, summary of CRISPR alleles). Students worked on their own initially and then in a group to compare answers.

**TABLE 2:** The effect of *CCR5* mutant alleles on reading frame and protein structure

Allele	Nucleotide change in coding sequence	Effect on reading frame (RF)	Effect on amino acids	Protein length	Protein structure
Unmodified *CCR5*		Normal (RF +1)		352	7 TM domains with extracellular N′ and intracellular C′
Delta 32 (Δ32) *CCR5*	Deletion of 32 nucleotides (911..942)	Frameshift (RF +3) leading to early stop codon	New amino acids on C terminus (31 different amino acids from AA185)	215	Lacks 3 TM domains, altered C′ protein sequence after TM 3
Lulu allele (Delta 15)	Deletion of 15 nucleotides (900..914)	In‐frame deletion	Deletion of 5 amino acids from AA181 in extracellular loop 2	347	Extracellular loop 2 missing 5 AA
Nana allele 1 (Delta 4)	Deletion of 4 nucleotides (913..916)	Frameshift leading to early stop codon (RF +2)	9 different amino acids on C terminus from AA186	194	Lacks 3 TM domains, altered C′ protein sequence after TM 3
Nana allele 2 (Plus 1)	Addition of 1 nucleotide at bp 911	Frameshift (RF +3) leading to early stop codon	42 different amino acids on C terminus from AA185(31 of the amino acids are the same as Δ32)	226	Lacks 3 TM domains, altered C′ protein sequence after TM 3

#### 2.3.2 *Comparative analysis of CCR5 gene and modified allele variants on computer*

Students were guided to obtain the full reference coding sequence of *CCR5* from NCBI (Accession number NM_000579). They created a “new sequence” in the DNA analysis software SnapGene. Students were tasked to create “new features” that represent the three exons to show where intron/exon boundaries would exist in genomic DNA. Using the open reading frame feature, the students determine the translated protein sequence and normal length of protein. They were given the three other new allele variants (Δ32, Lulu, Nana allele 1 and Nana allele 2) that had been created by the instructor based on reported CRISPR modified sequences.^32^ Students were guided to align these sequences together and determine the nucleotide, reading frame, amino acid sequence and protein length changes that were created. The use of the full version SnapGene is key in visualizing the alignment. Although NCBI and free DNA softwares ApE and Benchling enables alignment of sequences together, the alignment in Snapgene is presented in an effective scheme that enables students to still visualize the double‐stranded DNA molecule with polarity of each DNA strain, the resulting translation, and the features labeled in one window (Figure 2b,c, Figure S1). Students compared their analysis on SnapGene to their worksheet. Using SnapGene allows students to determine whether premature stop codons are formed further downstream from the nucleotide change.

#### 2.3.3 *Predicting the effect of the mutations on protein structure*

**FIGURE 3:**
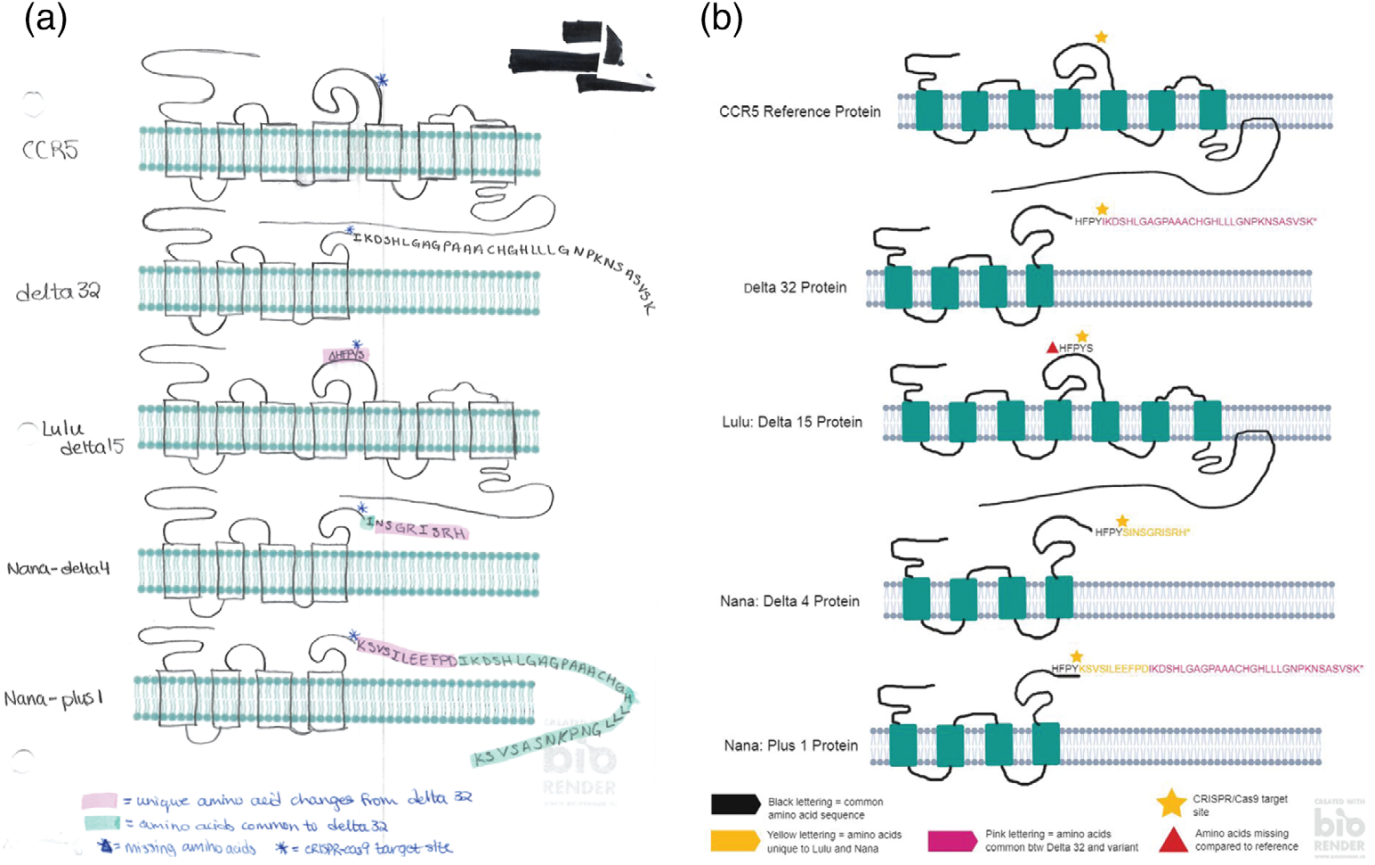
Student work depicting protein structure of CCR5 and predicted structures of its various modified versions (a) on paper and (b) using Biorender. Nucleotide sequences that were inserted or deleted are indicated, and nucleotides shared between Δ32 and Nana+1 alleles are highlighted

The nucleotide sequence alignment allows students to visualize the effect on the linear primary sequence of the protein, but it is important that students move beyond the linear protein sequence and establish the effects of changes in the primary sequence to the tertiary structure. Students were shown published tertiary structures of the CCR5 protein^33^, ^34^ where the unmodified protein consists of seven transmembrane helices, characteristic of all G protein‐coupled receptors. Additionally, the web‐based 3D structure viewer iCn3D can be used by students to visualize the tertiary structure of the protein. The students were tasked to discuss and predict how the new alleles affect protein structure and draw the various predicted protein structures out on paper. Students of the Advanced Genetics class utilized the software BioRender (https://biorender.com), a free biology drawing software to create their schematics. Examples of student work are shown in Figure 3a,b.

#### 2.3.4 *CRISPR‐cas9 editing design and strategy of CCR5 gene*

**FIGURE 4:**
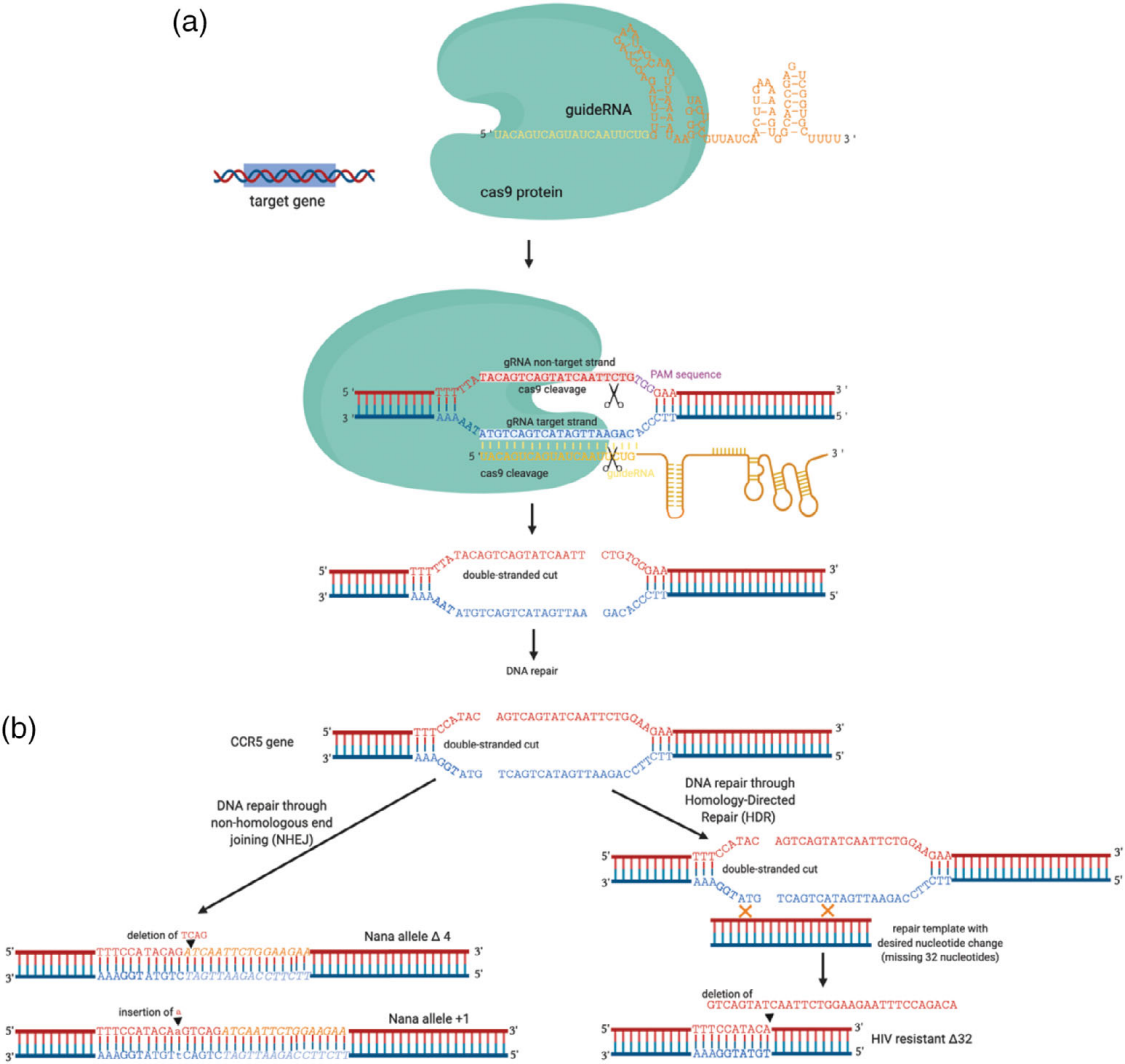
Necessary components for CRISPR‐cas9‐based genetic modifications. (a) cas9 and gRNA form a complex, then gRNA base pairs with complementary sequence in CCR5 gene with PAM aiding in bringing cas9 nuclease in, resulting in cas9 double‐stranded cleavage 3 nucleotides from PAM sequence. (b) A comparison of endogenous DNA repair mechanisms, non‐homologous end joining (NHEJ) and homology directed repair (HDR), which utilizes a repair template containing the desired nucleotide change. Nana alleles Δ4 and +1 are shown as outcomes of NHEJ, and HIV resistant Δ32 is shown as an outcome of HDR with the use of an appropriate repair template

Students were guided to design the strategy to program the cas9 nuclease to target and cut *CCR5* gene at the appropriate region in order to create a loss‐of‐function CCR5 protein. Specifically, students identified and designed 3 key components: (a) the cas9 nuclear target sequence in *CCR5*, (b) the protospacer adjacent motif (PAM) sequence in *CCR5* gene utilized by cas9, and (c) the complementary guide RNA that is guided to the target sequence by cas9 (Figure 4).

**FIGURE 5:**
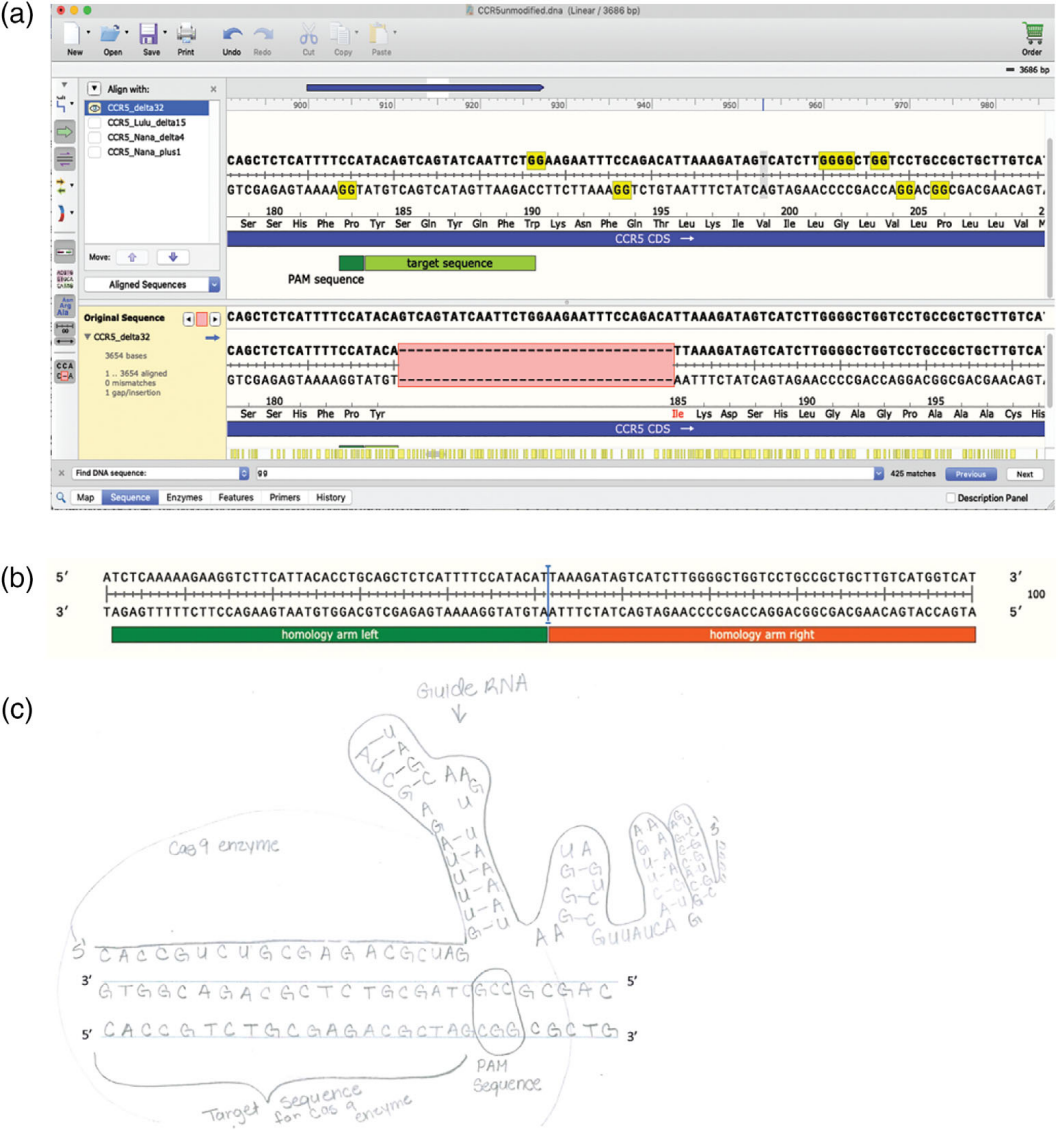
Key components utilized by CRISPR‐cas9 system. View in SnapGene of (b) PAM sequence (green) and gRNA‐cas9 target sequence (light green) are annotated in unmodified CCR5 allele in top half. Potential PAM sequences are highlighted in yellow. Bottom half shows the alignment with Δ32 allelic variant to allow visualization of which PAM sequence is most appropriate. (b) An example HDR repair template lacking the 32 nucleotides missing in the Δ32 allelic version, and containing two homology arms. The blue line indicates where the 32 nucleotides in Δ32 allelic version has been deleted. (c) Student work showing a schematic of gRNA and cas9 complexing with target sequence in *optix* gene

Using the reference unmodified *CCR5* gene, students utilized the “features” tool on SnapGene to identify and annotate the cas9 specific PAM sequence (5′NGG3′) chosen by Dr. He that was presented in his oral presentation at the Second International Summit on Human Genome Editing meeting (Start at: 1:17:57).^8^ The guanine dinucleotides of the PAM sequence that is on the non‐target strand of the gRNA interacts with arginine amino acids of cas9 to assist the unwinding of double‐stranded DNA for subsequence nuclease activity (as reviewed in Chen and Doudna^3^). Next, students were guided to label the target sequence of the cas9 nuclease, which are the 20 nucleotides upstream of the 5′TGG3′ PAM sequence (Figure 5a). The cas9 nuclease is brought to the target sequence by the single guide RNA, which is composed of an RNA sequence complementary to the target strand and an 80‐mer universal scaffold region that aids in cas9 binding. To model formation of gRNA‐cas9‐target DNA complexes, students were tasked to hand draw how all these components interact, labeling important components and sites including their PAM sequence, the location of the cas9 cut site, the annealing of the gRNA to its specific target nucleotide sequence. We intentionally had students draw this out based on our prior teaching experiences where assessment revealed students found it challenging to conceptualize the action and polarity of gRNA and cas9 complex with two complementary DNA strands (target and non‐target strands) of the target gene.^19^

Students were prompted to discuss why this specific PAM associated with the target sequence was chosen of all possible PAM sequences of (5′NGG3′) as a quick “Find” of “GG” highlights in yellow all the GGs present in the sequence (yellow boxes, Figure 5a), and students observe that there are many potential PAM sequences present throughout the gene. The discussion led the class to conclude that this specific 5′NGG3′ was chosen of many potential 5′NGG3′ because of the necessity for cas9 cut the *CCR5* genomic DNA in close proximity to the physical location of the desired nucleotide change that could potentially give rise to Δ32 allele variant.

#### 2.3.5 *Interpreting DNA repair mechanisms that created Nana and Lulu alleles; designing a homology directed repair (HDR) repair template*

Identification of the likely *CCR5* PAM sequence and cas9 cut site was followed by discussion of the outcomes for the *CCR5* gene after cas9 cleavage, and how different endogenous repair mechanisms are used to achieve different research goals. Animation are shown to illustrate the two processes.^35^, ^36^ The process of non‐homologous end joining (NHEJ) is used after cas9 makes the double stranded cut to produce indels and potential loss‐of‐function alleles due to frameshift mutations. Homology Directed Repair (HDR) is typically utilized when a specific nucleotide change is desired. HDR requires the addition of a repair template with homology arms that enables the cell to utilize HDR to incorporate a desired nucleotide sequence after the cas9‐mediated dsDNA cut. As a class, we discussed the features that the HDR must contain: (a) the desired nucleotide change, (b) regions of DNA that were similar to the original allele (homology arms), and (c) a PAM sequence that was altered such that the repair template would not be cut by cas9. Students were guided to create an HDR template in SnapGene (Figure 5b). Students were then asked to work in groups to discuss which DNA repair strategy was likely utilized by Dr. He to produce the three new variant alleles found in Nana and Lulu. Most students report that NHEJ occurred in the embryos to create the three new variants and that a repair template was likely not used as the HIV resistant Δ32 allele was not found in Lulu and Nana.

#### 2.3.6 *Putting knowledge into practice: CRISPR‐cas9 gene editing strategy with butterfly wing patterning gene optix and human oncogenic Ras gene*

Students worked on two follow‐up hypothetical scenarios to practice the CRISPR design and strategy of sgRNA and target sequence. The first scenario was that students were investigating the function of the gene *optix* in *Lepidoptera* butterflies, and were tasked to design a CRISPR‐cas9 strategy to create a loss‐of‐function *optix* gene. The students were shown published results of a CRISPR‐cas9 strategy, where *optix* CRISPR‐ed butterflies displayed color and patterning defects.^37^ An example of student work is shown in Figure 5c. The second scenario was that they had cultured cancer cells growing in lab with Ras gene that has the oncogenic mutation that causes abnormal cell proliferation.^38^, ^39^ The students' goal was to design a strategy to take this mutant Ras and genetically modify it to wild‐type Ras. They were given the coding sequence for the Ras gene in SnapGene with the oncogenic mutation causing 12th amino acid Glycine to Valine change (G12V). Students were asked to discuss in pairs what they would predict the phenotypic change would be after successful gene edit. The work was submitted to be checked by the instructor.

#### 2.3.7 *Bioethics discussion on implications of CRISPR‐cas9 technology and CCR5 editing in human society*

Following the exercise, we engaged the students in a robust bioethical discussion on the bioethical implications of CRISPR‐cas9 technology and the modification of the CCR5 gene. Questions that were posed to students for discussion are included in the Tables S2 and S3. One topic was the discussion of the novel and Δ32 mutations on CCR5's normal function in human health and disease. Whether Dr. He's novel *CCR5* mutations in the twins actually confer HIV protection or has an effect on normal immune function has not been published yet. We asked students whether Lulu and Nana should be monitored by a specialized group of doctors and researchers for unintended consequences of their genetic edits since they have novel modified alleles that have not been studied before. Since Dr. He's made his claim, studies show that humans who are carriers for the Δ32 *CCR5* allele have a faster recovery from strokes.^40^ Furthermore, *CCR5* plays a role in brain cognition, as mice that lack CCR5 have improved memory.^41^

## 3 RESULTS AND DISCUSSION

### 3.1 Student learning of CRISPR‐based concepts

To assess if students could effectively learn the basics of CRISPR‐based gene editing, without traditional “wet laboratory” bench‐based manipulations, we deployed a “dry lab” computer‐based module using human germline editing of *CCR5* as a case‐based learning study. This was implemented in two separate courses at a primarily undergraduate institution (PUI) in Spring 2018 semester. Courses were designed for mid to upper level science majors (300‐level Genetics course with 17 students, and 400‐level Advanced Genetics course with 11 students) with a vested interest in advanced topics in genetics and genetic engineering. In general, students were enthusiastic about studying CRISPR‐cas technology, especially using an example that was a very recent and controversial development in the field. Our learning objectives (Table 1) were framed around core components of CRISPR‐cas editing, focusing on the technical design and outcomes of gene editing concepts. To assess student achievement of these learning objectives, we chose not to formally assess student work on the *CCR5* gene, as this work was instructor guided. Instead, we assessed students on learning objectives in a post‐activity assignment where students could effectively showcase their knowledge (Table 3, Table S3/Rubric).

**TABLE 3:** Student learning objectives for application of CRISPR‐cas in research proposal

	Student learning objective	Means of assessment	Percent of students achieving LO
1	Explain gene chosen for modification and the justification for gene and desired change in cell/organism of choice	Introduction in research proposal	77.78%^a^
2	Describe the CRISPR‐cas9 strategy Determine appropriate target sequence and PAM Sequence in target gene Determine the gRNA sequence utilized Create repair template if HDR is utilized	Methodology in research proposal	100%^a^ 100%^a^ 100%^b^
3	Describe how the effect of the gene modification will be measured if gene modification is successful, and expected results and challenges	Methodology in research proposal	66.67%
4	Describe the bioethical implications of using CRISPR‐cas9 system in your system	Discussion in research proposal	55.56%

^a^ Nine groups of two students.

^b^ Eight groups of two students, one group did not choose to utilize HDR mechanism.

**FIGURE 6:**
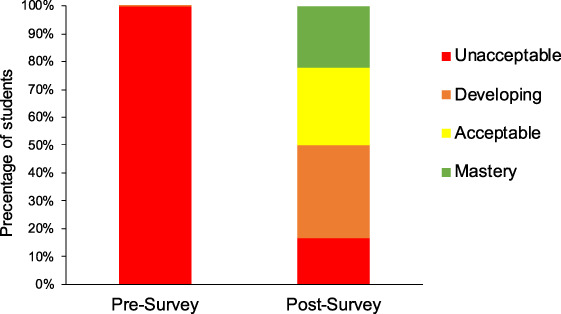
Student pre‐ and post‐survey responses to the question “Explain your current understanding is of how the process of CRISPR‐cas9 gene editing works. What are the molecular components, and how are they utilized by the system?”

Pre and post‐surveys asked students to “explain their current understanding of how the process of CRISPR‐cas gene editing works, and describe the molecular components, and how they utilized by the system.” In pre‐surveys, most answers were unacceptable (93.33%) or developing (0.07%), and in post‐surveys there is an increase in students' understanding of CRISPR‐cas9 technology with 50% achieving acceptable or higher (Figure 6). These results indicate that the use of a case‐based module is an effective strategy for disseminating the fundamentals of CRISPR‐cas‐based technology.

Our ultimate goal of this exercise is for students to then be able to apply their acquired knowledge, requiring them to demonstrate knowledge beyond simple comprehension and reiteration of facts. We therefore asked students to complete exercises demonstrating their ability to apply CRISPR‐related concepts to novel scenarios. Students in our 300‐level Genetics cohort were tasked to create a research proposal that asks a scientific question utilizing CRISPR‐technology in their organism of choice (Table 3). Specifically, students determined a gene of their choice that they would like to modify and proposed how they would phenotypically assay the success of their edit. Some students were initially overwhelmed by the open‐ended nature of the research proposal, but once they started investigated their gene of interest, they became more engaged. Student and faculty hesitation with novel research in courses are not uncommon; however, there is overwhelming evidence to suggest that once adopted, novel research is an effective teaching and learning practice.^42^, ^43^, ^44^, ^45^, ^46^

Students proposal topics and results of assessment are summarized in Table 4. Students were assessed on their justification of their gene and desired change, and 77.78% of students achieved acceptable or higher in their justification. Assessment showed that all students could describe their CRISPR‐cas strategy in their proposal through accurately determining appropriate target sequence and PAM sequence in desired gene, and designing the gRNA and repair template utilized to make their desired nucleotide change. This shows transference of knowledge from the *CCR5* exercise, and that students were able to master the technical aspects of CRISPR‐cas9 technology design. However, 66.67% of students achieved the learning objective of effectively describing how the effect of the gene modification would be measured and their expected results and challenges. Achieving this learning objectives requires higher order thinking in terms of the relationship between genotypes and phenotypes by determining and describing a valid assay to measure the success of their gene modification. All teams were also required to rationalize the bioethical implications of using the CRISPR‐cas system in their system of choice. 55.56% of the students could effectively describe the bioethical implications of using the technology in their system. Notably, only one team proposed a strategy to modify a gene in human embryos, while all other teams chose either human somatic cells or other organisms. Considering the required bioethical component of this proposal, this likely signifies that students may be unwilling to engage in such controversial dialog. In anything, student resistance highlights a need for bioethical training, even at the undergraduate level.

**TABLE 4:** Student proposed CRISPR‐cas target genes and strategies in research proposal

Target gene	Target organism	Strategy	Phenotype
Pax 6	*C*. *elegans*	Create loss‐of‐function Pax 6	Nervous system development
TRAK1	Human T‐cell	Create wild‐type version from a mutant version	Trak1 stability and GABA signaling
Dystrophin	Human muscle cells	Create wild‐type version from a mutant version	Gain in muscle mass and strength
CH1 gene encoding the Fel d 1 protein	Cat	Create loss‐of‐function CH1	Reduction in human allergies to cat
Hairless (HR)	Naked mole rat	Create specific nucleotide change to cause tryptophan to cysteine change	Hair growth
Opsin	Mice	Create loss‐of‐function opsin	Reduce blue color vision
tga1	Maize	Create specific nucleotide change (to cause asparagine to lysine change)	Increase in encased corn kernels (teosinte‐like phenotype)
KIAA0319	Human embryos	Create specific nucleotide change of T to C; changing a SNP associated with dyslexia	Change in dyslexia symptoms
CFTR	Human lung cells	Inserting 508th amino acid that is deleted in CFTR patients	Reduction in CFTR symptoms

In the 400‐level advanced genetics course, students worked in groups to create a podcast for the general topic focused on a theme of CRISPR‐cas technology. We chose to create podcasts over research proposals for two main reasons. Firstly, one of the course goals was to develop student communication skills, and secondly, this exercise was used intentionally in the first weeks of Advanced Genetics as an introduction into the rest of course material focusing on current findings and applications of CRISPR‐cas technology through primary literature. The three topics chosen for each group's podcast included: (a) CRISPR‐cas history, guidelines, and treatment of disease; (b) CRISPR‐cas gene editing in human embryos and live humans; and (c) CRISPR‐cas agriculture and food. (Rubric for the podcast, Table S4). When assessed for content and depth of knowledge, student work again demonstrated that fundamental aspects of CRISPR‐mediated gene edits were being understood by students (data not shown). Again, these data signify that learning modules such as the one herein is an effective and engaging means to teach these concepts to undergraduates.

### 3.2 Case‐based learning modules as gateway into authentic research practices

Bringing CRISPR‐cas9 technology to undergraduate curriculum can prove challenging especially in circumstances where resources or instructor familiarity might be limiting factors. Although hands‐on experimentation with CRISPR systems is not feasible for every institute, we tested whether a case‐based learning module is sufficient to teach students about the effective use of CRISPR‐cas technology in authentic research. We asked students to report the effectiveness of using CRISPR‐cas technology in (a) learning how authentic biology research is conducted, and (b) their abilities to apply tools learnt in experimental research and design (Figure S2a,b). The majority of students who did not see the importance of these examples before engaging in the exercise could clearly see the relevance upon completion of the module. These data suggest that students found they could apply this case‐based module to authentic practices in the research process.

Although this module is mainly computer‐based, the manipulation of gene sequences using basic molecular biology software (SnapGene) proved to be valuable to the student learning experience. Recent findings have shown that computer‐based undergraduate research experiences in lab courses produce similar learning outcomes and higher sense of achievement and satisfaction as bench‐based lab experiences.^47^ This suggests computer‐based lab experiences can have positive effects on students' mindset.

Anecdotally, students have difficulty visualizing the design, implementation and expected results of manipulating sequences. Here we took a backwards design approach, working from affected protein produced from CRISPR‐cas‐based edits to the unmodified *CCR5* gene for students to the visualize how their desired outcome necessitate thinking critically about each process of the central dogma. This technical design can be done without the use of sequencing computer software; however, we find that the use of SnapGene (or similar software) is helpful in allowing students to conceptualize how changes at the genomic level affect the gene product. When asked students for written anonymous feedback about the use of SnapGene to support our lab learning outcomes, students commented on the effectiveness of visualizing nucleotides, amino acids and features easily. Additionally, the ability to make desired nucleotide changes to simulate gene edits/modification was beneficial. A few students did comment on the challenges of learning how to use the program initially, but that with practice they were able to use the program more effectively. We should also note that student understanding seemed to be drastically improved when they could visualize both strands of the DNA and its polarity, not just coding strand of the desired target.

### 3.3 Beyond case‐based learning modules—Expanding into other high impact practices

In the future, we can imagine instructors expanding this case‐based learning module focused on *CCR5* into other areas of biology and medicine. For example, bringing into class a published example where students can analyze data from an individual with HIV and leukemia was given a stem‐cell transplant from a donor homozygous for *CCR5* Δ32.^48^ As a result, genotyping and phenotypic analysis revealed the individual had switched to homozygous Δ32 genotype and was cured of HIV and leukemia and HIV. This allows for deeper discussions into experimental data that probes the relationship between genotypes and phenotypes.

In our iteration of this module in Advanced Genetics, complementing this exercise with primary literature led us to analyze and discuss methodology and data of two scientific papers. The first paper was used as a historical perspective of what had been done previously in the field using Zinc Finger Nucleases to genetically modify T cells of individuals to Δ32 version and then transplant back into an individual (autologous transplantation).^49^ As a class we identified the Zinc Finger Nuclease target sites within the *CCR5* gene. The second paper was used to discuss how scientists have used CRISPR‐cas9 technology to create indels in *CCR5* of T cells through NHEJ and test its effect on HIV infection.^50^ Following the focus on *CCR5*, we went on to study the recent work on gene editing of *dystrophin* gene in muscle dystrophy in dogs, and continued to use SnapGene to analyze CRISPR genes.

This module can also be used as a framework to create additional case‐based learning studies for different diseases that are currently being targeted with somatic, non‐heritable/germline CRISPR‐cas technology. For example, currently the first human clinical trial utilizing CRISPR technology for immunotherapy cancer treatment is underway at University of Pennsylvania by removing individual T cells, modifying genes of T cells in lab, and then putting back the modified T cells into individuals to attack cancer cells.^51^, ^52^ Other current examples include, companies working to modify the *BCL11A* gene and beta‐globin gene to treat inherited blood disorders beta thalassemia and, sickle‐cell disease^53^, ^54^, ^55^ (Vertex, Editas, Sanford University). These “ex vivo” methodologies involve a different set of considerations, both from the scientific and ethical perspectives. A notable case is the first human in vivo CRISPR editing clinical trial called EDIT‐101 at the genome editing company Editas. Editas has received FDA approval for utilizing CRISPR technology to modify the *CEP290* gene in photoreceptor cells to treat retinal degeneration condition called Leber Congenital Amaurosis 10.^56^, ^57^, ^58^ The methodology differs here because scientists will use CRISPR technology and adeno‐associated viral (AAV) delivery system to deliver CRISPR components to edit genes inside the human body in vivo rather than ex vivo. Collectively, these different studies and clinical trials can be used to immerse students in analyzing biomedically relevant genes and associated diseases in the classroom, and bring forward the discussion on how both non‐heritable and heritable CRISPR gene editing is being designed and applied in basic science and medicine.

## CONFLICT OF INTEREST

The authors declare no potential conflict of interest.

## Supporting information


**Appendix** **S1**. Instructor guide/key.

**Appendix** **S2**. Student worksheet.

**Appendix** **S3**. Figures S1, S2 and Tables S1 to S5.
**Figure S1**. Alignment of various *CCR5* alleles against unmodified *CCR5* allele. Top window shows the unmodified *CCR5* allele, and the bottom window shows the alignment with (a) Δ 32 allele, (b) Nana +1 allele, (c) Nana Δ4 allele and (d) Lulu Δ15 allele. Red boxes highlight mismatches, and red asterisk indicates stop codon that resulted from frameshift.
**Figure S2**. Student pre‐ and post‐survey self‐reports on the (a) effectiveness of CRISPR‐cas9 technology in learning how authentic biology research is conducted and (b) ability to apply tools learnt to experimental research and design.
**Table S1**. Bioethical Discussion Question on CRISPR germline editing
**Table S2**. *CCR5* CRISPR‐cas gene editing‐specific Discussion Questions
**Table S3**. Rubric for Assessing Student Learning Objectives in Research Proposal
**Table S4**. Rubric for Assessing Student Learning Objectives for Podcast Assignment
**Table S5**. Useful links
