# Supplementary material for: “Designer babies?!” A CRISPR‐based learning module for undergraduates built around the CCR5 gene
Source: Biochem Mol Biol Educ. 2020 Aug 10;49(1):80–93. doi: 10.1002/bmb.21395 (PMC7891609; doi:10.1002/bmb.21395)
Supplement: Supplementary file 2 — Appendix S2. Student worksheet. [file BMB-49-80-s002.docx]

**Student Learning Outcomes for this case-study:**

**By the end of this class, students will-**

**1) link principles of DNA, RNA and protein**

**2) determine how reading frame, amino acid sequence is affected by CRISPR-cas technology based gene edits**

**3) visualize DNA sequence on DNA sequence analysis software Snap Gene**

**4) annotate guide-RNA/cas9 target sequence and PAM sequence in CCR5 sequence**

**5) model and draw gRNA/cas9 activity on target DNA sequence**

**6) design a homology-directed repair (HDR) repair template used in DNA repair after gRNA/cas9 activity**

**Background:**

In preparation for this class, you read the MIT technology review article: “Exclusive: Chinese scientists are creating CRISPR babies”.

<https://www.technologyreview.com/s/612458/exclusive-chinese-scientists-are-creating-crispr-babies/>

and watched the youtube video: <https://www.youtube.com/watch?v=th0vnOmFltc>


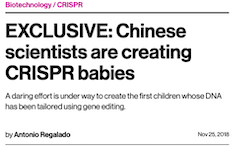

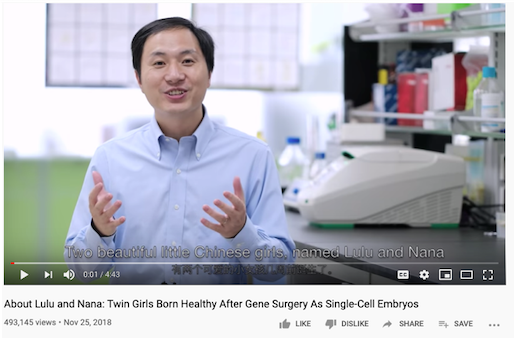


A team of scientists, led by Dr. He Jiankui, claimed to have modified the gene called *CCR5* in two twin girls using a gene editing technology called CRISPR-cas9 technology. This raised an international response about the bioethical implications of gene editing in human embryos. Today we will analyze the gene sequences modified in the twin girls, the effect the changes have on protein structure and function, and using a sequence software on the computer, model how Dr. He designed his CRISPR experiment

***Part 1 -* *Comparative analysis of CCR5 gene and modified allele variants on paper*:**Below are coding DNA sequences shown by Dr. He at the 2^nd^ International summit on human gene editing on DNA sequence data he collected on the embryos of the twin girls. On the following page is the DNA sequence for the “unmodified *CCR5*” allele, compared to Delta 32 (Δ32), which is a naturally occurring allele found in the human population that shows resistance to HIV viral infection. The three sequences that follow are CCR5 alleles in the two twin girls Lulu and Nana that were CRISPR modified by Dr. He.

Use the codon chart provided by your instructor.

**Determine the**

- **reading frame**
- **the RNA transcript produced**
- **the change in protein sequence, what type of mutation(s) result?**

*Note* AGA is the first codon shown here; … indicated other nucleotides not shown.* **Unmodified CCR5:**

5’-…AGATCTCAAAAAGAAGGTCTTCATTACACCTGCAGCTCTCATTTTCCATACAGTCAGTATCAATTCTGGAAGAATTTCCAGACATTAAAGATAGTCATC

**Delta 32 (Δ32):**

5’-…AGATCTCAAAAAGAAGGTCTTCATTACACCTGCAGCTCTCATTTTCCATACATTAAAGATAGTCATC

**Unmodified CCR5:**

5’-…AGATCTCAAAAAGAAGGTCTTCATTACACCTGCAGCTCTCATTTTCCATACAGTCAGTATCAATTCTGGAAGAATTTCCAGACATTAAAGATAGTCATC

**Twin 1-Lulu:**

5’-…AGATCTCAAAAAGAAGGTCTTCATTACACCTGCAGCTCTCAGTATCAATTCTGGAAGAATTTCCAGACATTAAAGATAGTCATC

**Unmodified CCR5:**

5’-…AGATCTCAAAAAGAAGGTCTTCATTACACCTGCAGCTCTCATTTTCCATACAGTCAGTATCAATTCTGGAAGAATTTCCAGACATTAAAGATAGTCATC

**Twin 2-Nana Allele Variant #1:**

5’-…AGATCTCAAAAAGAAGGTCTTCATTACACCTGCAGCTCTCATTTTCCATACAGTATCAATTCTGGAAGAATTTCCAGACATTAAAGATAGTCATC

**Unmodified CCR5:**

5’-…AGATCTCAAAAAGAAGGTCTTCATTACACCTGCAGCTCTCATTTTCCATACAGTCAGTATCAATTCTGGAAGAATTTCCAGACATTAAAGATAGTCATC

**Twin-2 Nana Allele Variant #2:**

5’-…AGATCTCAAAAAGAAGGTCTTCATTACACCTGCAGCTCTCATTTTCCATACAAGTCAGTATCAATTCTGGAAGAATTTCCAGACATTAAAGATAGTCATC

***PART 2. Comparative analysis of CCR5 gene and modified allele variants on computer***

Requires: SnapGene, a DNA sequence analysis software, that allows visualization of DNA sequences. You can download a free 30-day trial of the full version at <https://www.snapgene.com/try-snapgene/>

The scientist Dr. He that claimed to have make a genetic change in twin girls tried to target the gene *CCR5.* It encodes for a receptor protein that is utilized by the HIV virus to enter target immune cells.

**1.** First let’s obtain the sequence for CCR5.

**2.** Go to: <https://ghr.nlm.nih.gov/gene/CCR5> to find out more information about CCR5

**3.** Click NCBI Gene. Scroll down


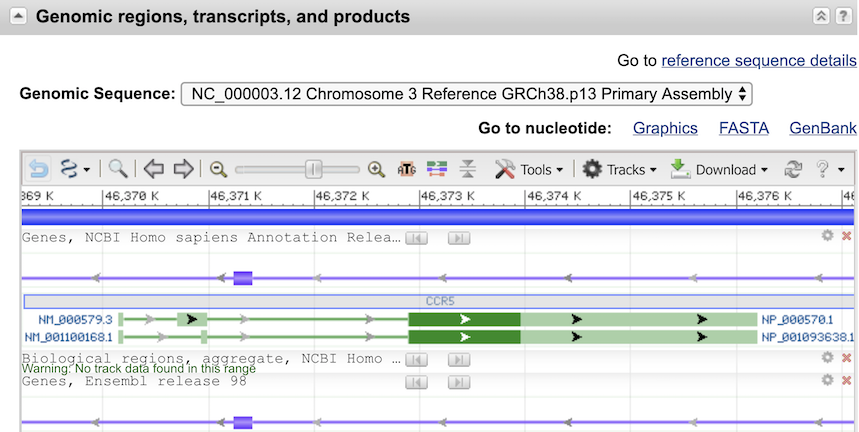


**4.** Hover over the green box with CCR5 label on top. (Don't hover over CCR5 label itself)

A gray box should appear:


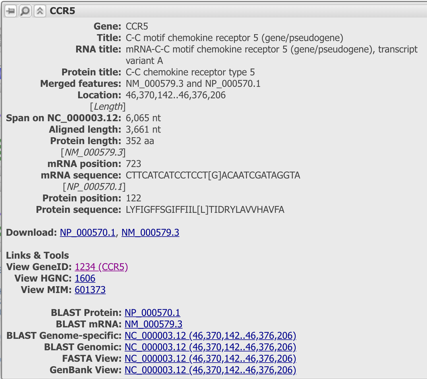


**5.** Look for the download box.

Next to the download box, there are two codes: NP…. And NM..

NP is a protein sequence and NM is a nucleotide sequence.

**6.** Download the NM nucleotide sequence onto your computer by clicking the NM code.

**7.** Open the file in SnapGene. Notice how you see both strands of DNA, and the 5’ and 3’ of each strand.

**8.** If we open the url for this nucleotide sequence: <https://www.ncbi.nlm.nih.gov/nuccore/NM_000579>

We can find out that the coding sequence is from base pair: 358..1416

***What nucleotides make up the following Exons?***

Exon 1: __________

Exon 2: ___________

Exon 3: ___________

**Annotating the *CCR5* gene**

We can annotate (label) the coding sequence (the sequence that encodes for the protein) by doing the following:

**9**. Highlight from base 358-1416, click on Features at the toolbar on top, then click on Add Feature.

A box will pop up, label the feature “CCR5 CDS” in the box, change Type to “CDS”, and click the option for “Translate this feature in Sequence view”.
You can change the color from Gray to color of your choice. Click “OK”.


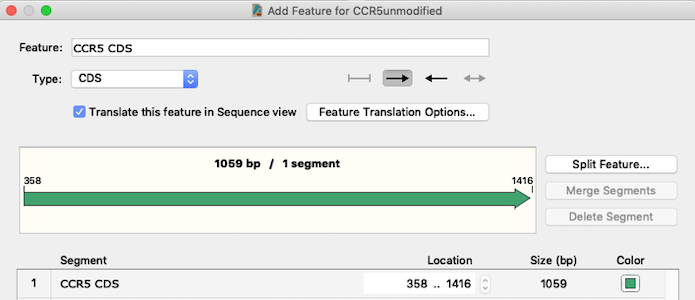


This will take you back to your sequence, and you can scroll to the right, and you should see your annotated coding sequence (CDS) labeled with the amino acids labeled as well with amino acid numbers.

***What is the first amino acid labeled?*** _________


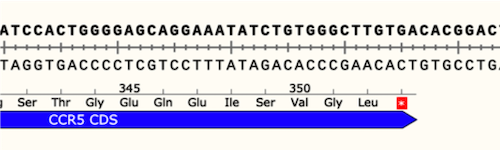


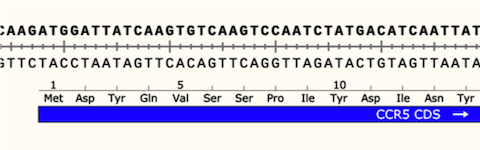


***What do you see at the end of the coding sequence?*** You should see a red asterisk, indicating a Stop codon. From the amino acid numbers you can determine ***how many amino acids make up the protein***: _________ amino acids with the last amino acid ______

**10.** You can also create features for each exon, by following the same steps.

**Alignment of unmodified reference sequence to Δ32 and CRISPR allele variants**.

**1**. From Blackboard download the three SnapGene files:

- Delta 32 (Δ32)
- Lulu Allele 1
- Nana Allele 1
- Nana Allele 2

**2.** To align:
Click Tools

Align to Reference Sequence

Choose the 4 files that you have downloaded

You will see on your screen your window split into two windows (as shown below)
In the top window you see the reference unmodified CCR5 gene. In the bottom window you will see, the alignment of the other sequences.

**
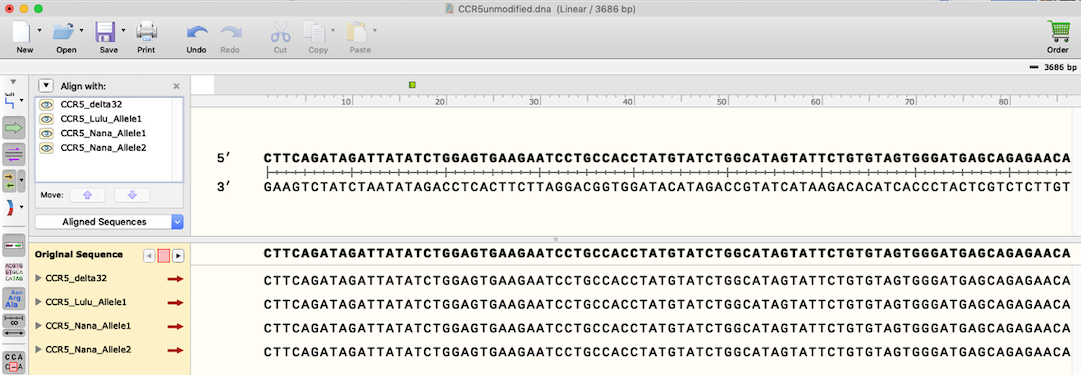
**

**3**. You can use your mouse to scroll the sequences from left to right, or right to left.

Look to see where there are nucleotide differences. Any difference you see will be highlighted in Red. ***Do you see any nucleotide differences?***

You should see an alignment that looks like this:


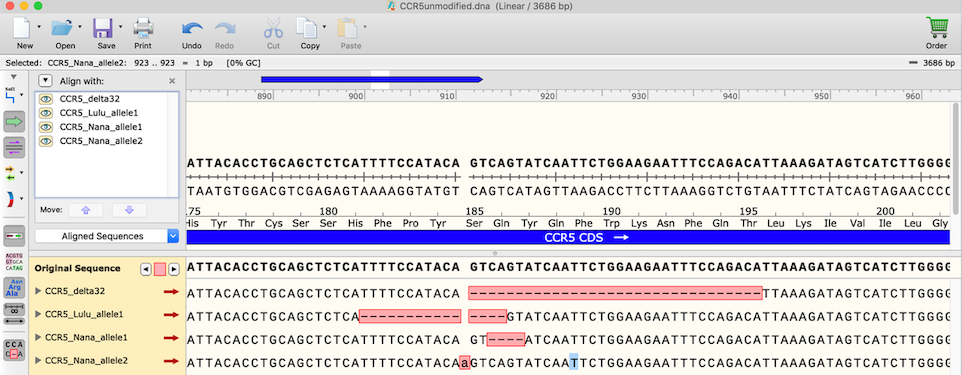


Dashed lines indicated nucleotides that are missing.

***Which allele shows an insertion of a nucleotide? __________________***

**4.** If you click on the triangle to the left of the sequence name in the yellow box, it will allow you to expand the nucleotide view. You should now see the two strands of the double-stranded DNA molecule, and the change in amino acid sequence.
Notice how for the Δ32 allele, after the deletion of the 32 nucleotides (highlighted in red) the amino acid sequence changes due to the change in reading frame, and an early stop codon (codon 216, indicated in red asterisks) is shown, showing that the protein will now be 215 amino acid in length.


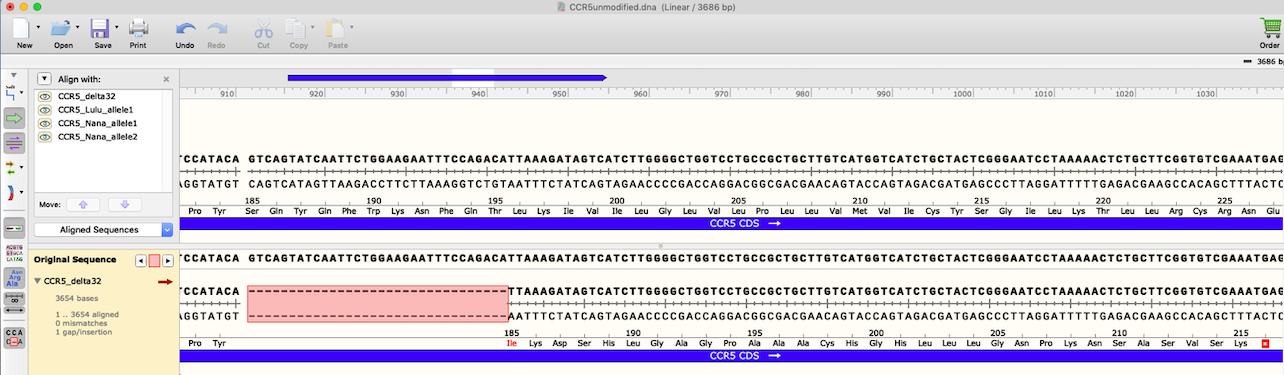


**5.** Determine how the nucleotide changes in Lulu’s and Nana’s alleles affect the reading frame and amino acid sequence.
***Does it match what you found on paper earlier in this exercise? Are there any early stop codons?***

***PART 3. Predicting the effect of the mutations on protein structure***

The CCR5 protein is a receptor protein residing in the membrane as shown below and as see in the animation of HIV infection.
<http://scienceofhiv.org/wp/?page_id=20>

From: Textbook, Microbiology the Human Experience, Foster et al.

**
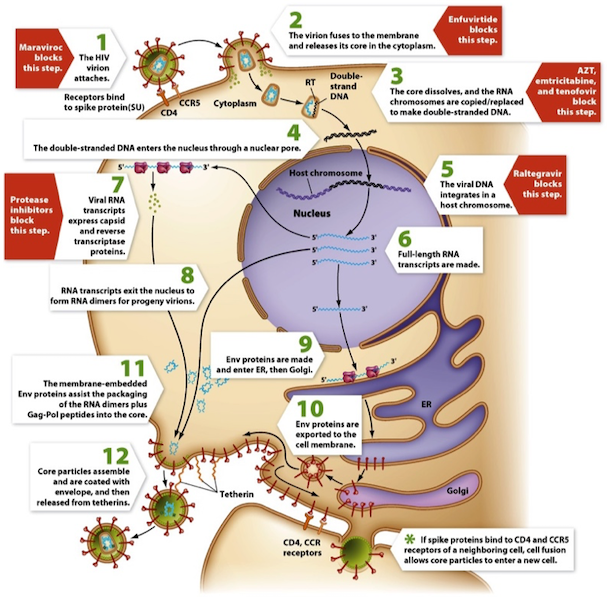
**

**When does the CCR5 protein interact with the HIV virion during HIV infection?**

The following is a representation of the tertiary structure of the protein. Each letter represents an amino acid.

***How many transmembrane domains does CCR5 protein have?*** ______________


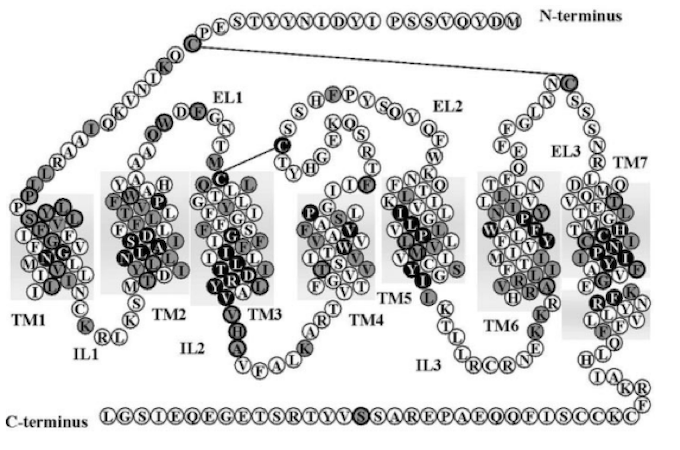

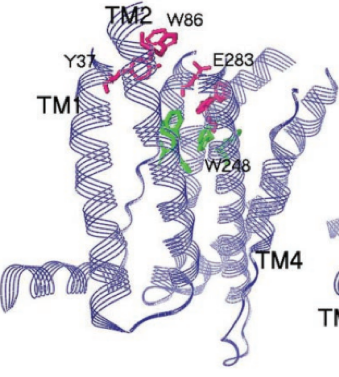


**1.** Let’s label the transmembrane (TM) domains encoded by our DNA sequence in our sequence file for the unmodified CCR5 allele. Using the features tool, label the different TM domains:

TM1: amino acid (AA) 32-58

TM2: AA 64-91

TM3: AA 102-128

TM4: AA 134-165

TM5: AA 193-220

TM6: AA 232-258

TM7: AA 276-299

**2.** Predict the nucleotide changes in the alleles affect the protein structure. Draw a simple schematic depicting the change in each of the proteins produced by the alleles. **How do you think the nucleotide changes affect the protein structure?**

For example, for the **unmodified CCR5 protein:**


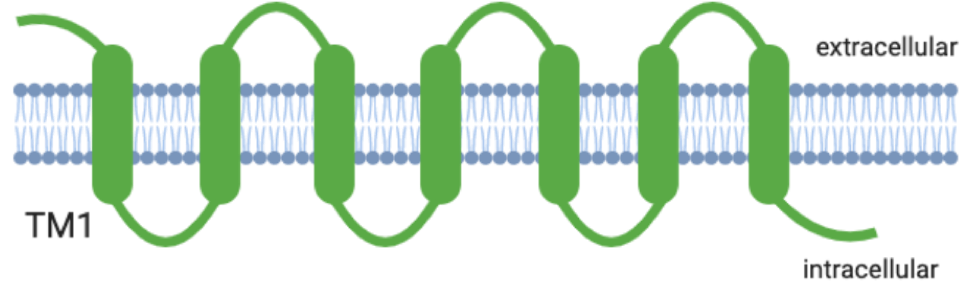


**Δ32 CCR5 version:**

***
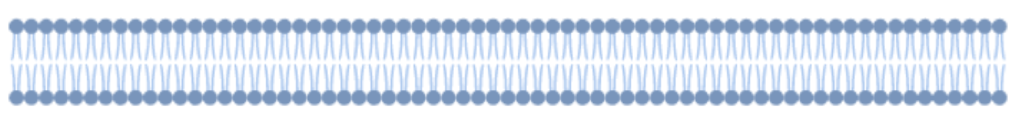
***

***Describe how you think the TM domains are affected in the Δ32 protein? __________________________***

Researchers have found that the Δ32 CCR5 protein is present in high frequency in Caucasian populations, and the 32-base pair deletion results in a non-functional receptor protein that does not allow proper HIV infection, thus allowing T cells to be resistant to HIV infection. ***How could a loss of TM domains lead to a non-functional receptor protein during HIV infection?***

**Lulu CCR5 - CRISPR edited version in Lulu:**

***
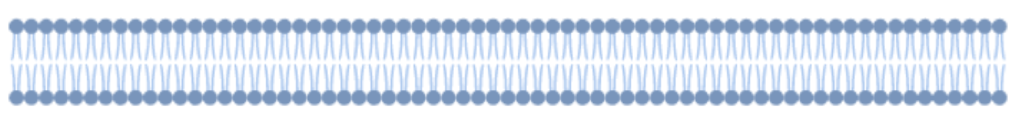
***

**Nana CCR5-1 - CRISPR edited version from Nana allele 1:**

***
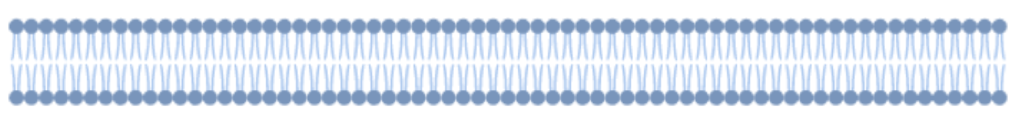
***

**Nana CCR5-2 - CRISPR edited version from Nana allele 2:**

***
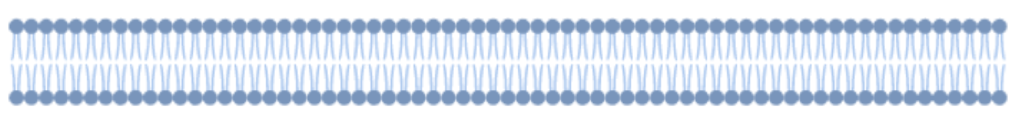
***

***Part 4. CRISPR-cas9 Editing Design and Strategy of CCR5 gene***

At the Second International Summit on Human Genome Editing: Continuing the Global Discussion 2019 <http://www.nationalacademies.org/gene-editing/2nd_summit/> the scientist Dr. He presented these edited sequences during his presentation claiming he has “CRISPR gene edited” the *CCR5* gene in the twin girls.

***
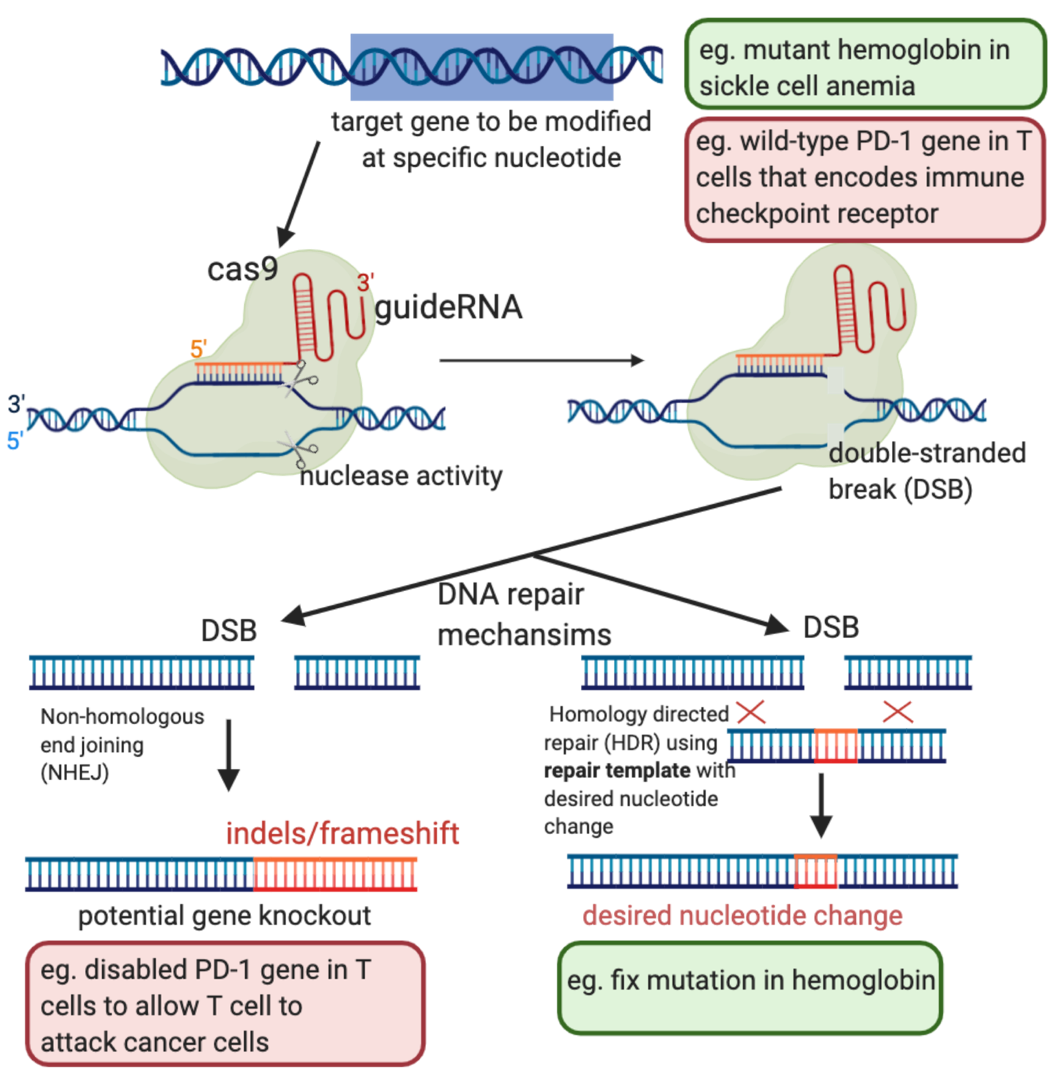
So how did he make these CRISPR edits?***

He used an enzyme called **cas9** (green, in schematic). cas9 is an endonuclease enzyme that acts like a “molecular scissors” to cut at a specific DNA sequence because cas9 is “guided” to the specific sequence by a “guide” molecule” called **guideRNA (**orange/red molecule in schematic). cas9 and the guideRNA form a complex together. The guideRNA is a short RNA molecule of about 100 nucleotides, and at its 5’ end, contains a unique 20 nucleotide sequence (orange, in schematic on the right) that is complementary to the target DNA (in blue). Because the 20-nucleotide sequence of the gRNA can hydrogen bond with the complementary DNA, scientists can change the 20-nucleotide sequence of the guideRNA to target specific DNA of interest, thus allowing cas9 to be “**programmed”** to target and cut at a desired sequence in DNA, which makes this complex a powerful tool.

Let’s watch an animation of this.

<https://media.hhmi.org/biointeractive/click/CRISPR/>

***What happens after the cas9 cuts the DNA? How can the double-stranded DNA break lead to specific gene edits?***

When cas9 cuts in a gene at the specific sequence, **a double-stranded break occurs**. This is followed by **two potential cellular DNA repair mechanisms** as the cell contains enzymes that repair double-stranded breaks in DNA:

1. **Non-homologous end-joining (NHEJ):** the two ends of the double-stranded break will be stitched back together. This is an error-prone process, thus there may be either mutations that occur or a loss of nucleotides, which can lead to a frameshift in the reading frame resulting in a change in coding sequence and/or early stop codon, which will inactivate the gene.
2. **Homology-directed repair (HDR):** is a less error prone process, where a homologous (similar) DNA template is used to more accurately repair the break, for example from a sister chromatid. Scientists will manipulate this process by inserting into the cell, a short DNA molecule that contains your nucleotide of interest and the sequence of the cleavage site, called a Repair Template, and the cell will be “tricked” into using the repair template to fix the DNA break. Scientists can design repair templates with to have desired nucleotides so that they can change the target DNA sequence into a new, desired sequence. For example, if there is a mutation in the target gene, scientists can design a gRNA to target cas9 to cut by the mutation, and then use a repair template to replace the mutation with a nonmutated nucleotide.

***Let’s determine where Dr. He targeted cas9 to cut in the CCR5 gene.***

In one of his slides during his presentation, he showed the *CCR5* sequences in the human embryos that he had CRISPR edited and sequenced. Each colored peak represents a different nucleotide, shown on the top as a sequence


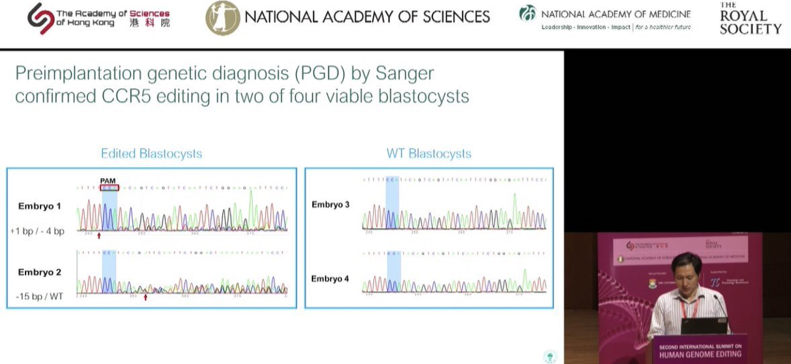


Notice how there is a sequence highlighted in his slide called a **PAM sequence**?

***What is the PAM sequence?*** It functions as a binding signal for cas9.
For cas9 to cut at the desired target sequence, a three-nucleotide sequence 5’-NGG-3’, where N is any nucleotide, must be next to the 20-nucleotide target sequence. This 5’-NGG-3’ is called a **Protospacer Adjacent Motif (PAM) sequence (purple, in schematic)**. The guanine dinucleotides of the PAM sequence interact with arginine amino acids of cas9 to assist the unwinding of double-stranded DNA and subsequent nuclease activity of cas9 once the gRNA binds to the target sequence. **cas9 cuts 3-4 nucleotides from the PAM sequence.**


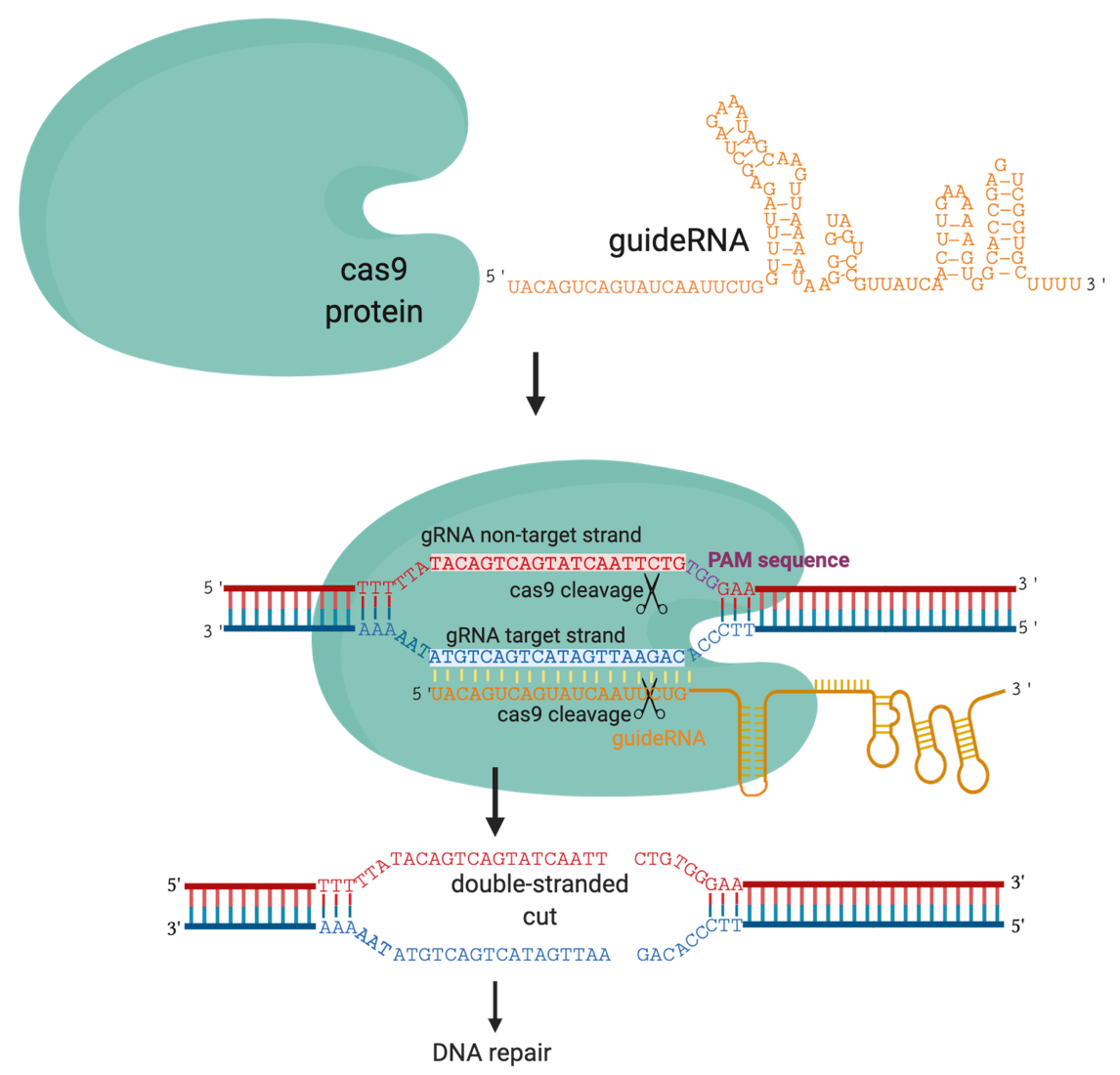


**Structure of cas9 enzyme with bound guideRNA and target DNA sequence.**

cas9 forms a complex with guideRNA. 20 nucleotides of the guideRNA anneals to the complementary DNA strand. PAM sequence aids cas9 binding. cas9 has two nuclease domains and cleaves 3-4 nucleotides from the PAM sequence on both DNA strands, leading to double-stranded break and DNA repair mechanism.

**1.** Let’s label the PAM sequence that Dr. He used in the unmodified *CCR5* allele in SnapGene.

**2.** Highlight nucleotide 904..906. In the left hand-corner you can see which nucleotide # you have selected.

**3.** Go to “Features”> Add Features.

Label the feature, PAM. You can change the color if you wish in the Color box. Hit OK.

**4.** The PAM sequence has the sequence 5’-NGG-3 where N is any nucleotide.
***Which strand (top or bottom) do you see the 5’-NGG-3’?*** __________________

**
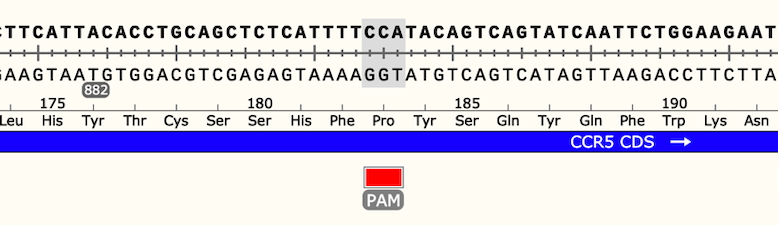
**

3’

5’

**5**. Let’s determine where the 20 nucleotide target sequence for the guideRNA is. Remember that the guideRNA is complementary and antiparralel to the target sequence. Use the figure from the previous page as reference.

***Should it be to the right or to the left of the 5’-TGG-3’ PAM sequence? _____________***

**6.** Label the target sequence in SnapGene. Using the features tools label the target sequence of 20 nucleotides


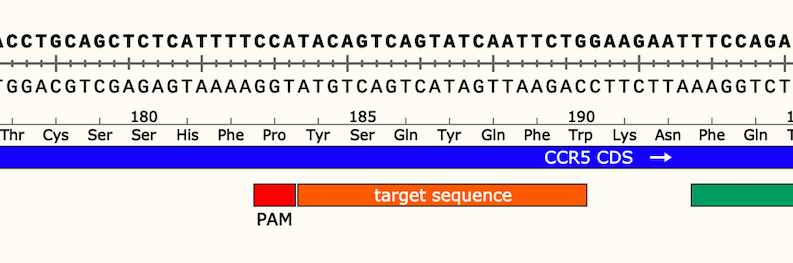


5’

3’

**7. Draw out below how the complementary guideRNA would bind to the target DNA and where cas9 would potentially cut the two strands of DNA.**

- **What is the sequence of the 20 nucleotide guideRNA that is complementary to the target DNA?**
- **Which strand, top or bottom, would the gRNA hydrogen-bond with? _______________**

**8.** In SnapGene, if you go to Edit> Find DNA Sequence> and type in GG, you will see there are lots of potential 5’-NGG-3’ PAM sequences highlighted in yellow.

**
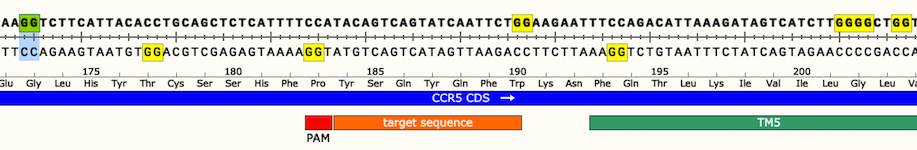

*Why do you think Dr. He chose this target sequence (and PAM sequence) to program his guideRNA/cas9 complex?***

5’

3’

***PART 5. Interpreting DNA repair mechanisms that created Nana and Lulu alleles; Designing a Homology Directed Repair (HDR) Repair Template***

**Discuss the following two questions with your table.**

**1. Did Dr. He end up making the desired HIV-resistant** Δ**32 allele variant in twins Lulu and Nana?**

**2. Which DNA repair mechanism do you think was utilized by the cell after the cas9 created the double-stranded cut?**

**3.** If Dr. He were to use HDR, he would have had to create a repair template containing the desired Δ32 nucleotide deletion and sequences that are homologous to the target gene at the site of cas9 cut (called homology arms). **Let’s create a repair template in SnapGene**. The length of the repair template can vary depending on cell type from 50 nucleotides to 2000 base pairs. For the purpose of our class, we will make a 100 base pair repair template.

**4.** We will use the Δ32 sequence from the alignment window. The desired nucleotide change should be in the middle, so we will go 50 nucleotides from the 5’ end of the Δ32 deletion/change to the left.
Copy that sequence and in a new DNA file (File>New DNA>), paste the 50 nucleotide sequence.
Then highlight and copy 50 nucleotides from the 3’ end of the Δ32 deletion/change and add that to the new DNA file at the 3’ end of the sequence. **You have created a repair template that the cell could be used to create the Δ32 variant.**

**5.** Scientists sometimes alter the PAM sequence in the repair template from 5’-NGG-3’ to a different sequence. ***Why do you think scientists would do this?***
