# Supplementary material for: “Designer babies?!” A CRISPR‐based learning module for undergraduates built around the CCR5 gene
Source: Biochem Mol Biol Educ. 2020 Aug 10;49(1):80–93. doi: 10.1002/bmb.21395 (PMC7891609; doi:10.1002/bmb.21395)
Supplement: Supplementary file 3 — Appendix S3. Figures S1, S2 and Tables S1 to S5. Figure S1. Alignment of various CCR5 alleles against unmodified CCR5 allele. Top window shows the unmodified CCR5 allele, and the bottom window shows the alignment with (a) Δ 32 allele, (b) Nana +1 allele, (c) Nana Δ4 allele and (d) Lulu Δ15 allele. Red boxes highlight mismatches, and red asterisk indicates stop codon that resulted from frameshift. Figure S2. Student pre‐ and post‐survey self‐reports on the (a) effectiveness of CRISPR‐cas9 technology in learning how authentic biology research is conducted and (b) ability to apply tools learnt to experimental research and design. Table S1. Bioethical Discussion Question on CRISPR germline editing Table S2. CCR5 CRISPR‐cas gene editing‐specific Discussion Questions Table S3. Rubric for Assessing Student Learning Objectives in Research Proposal Table S4. Rubric for Assessing Student Learning Objectives for Podcast Assignment Table S5. Useful links [file BMB-49-80-s003.docx]

A.

 
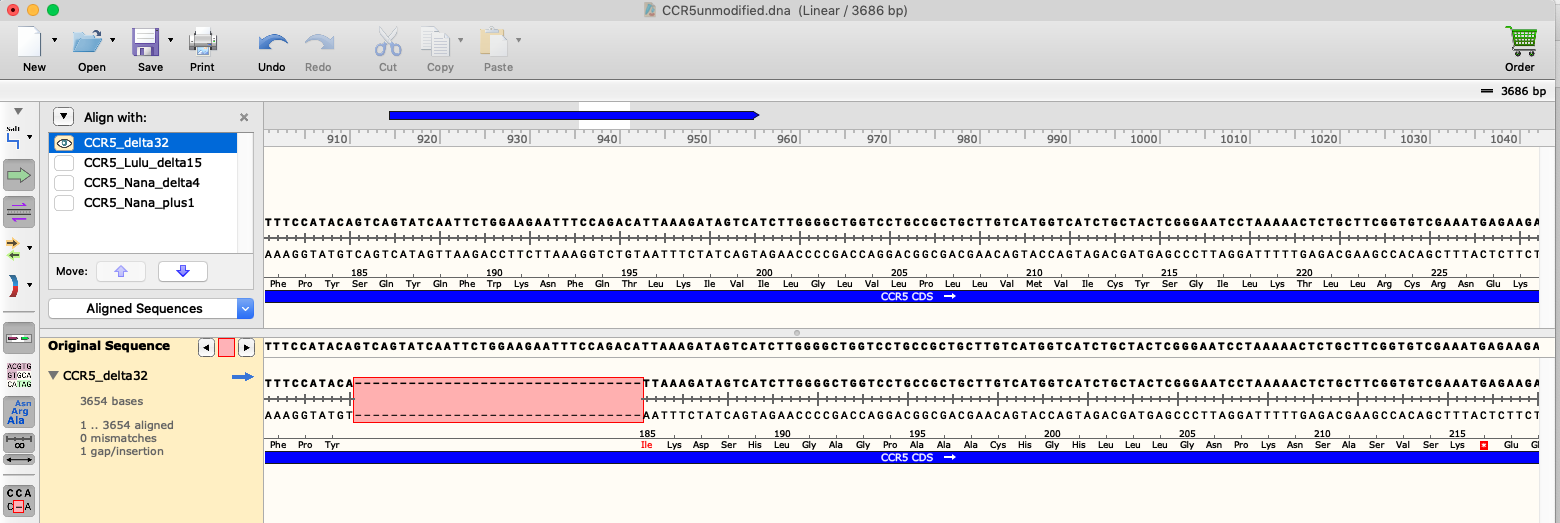


B.


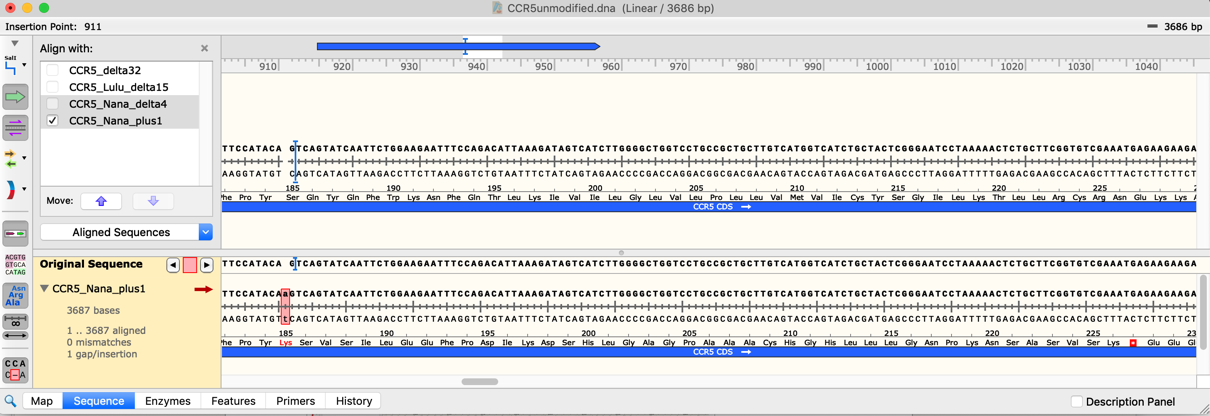


C.

_
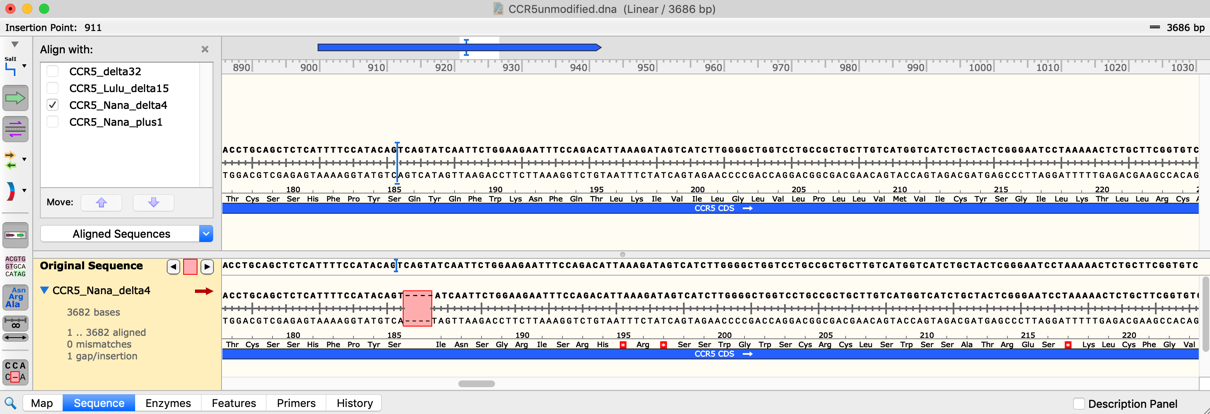
_

D.


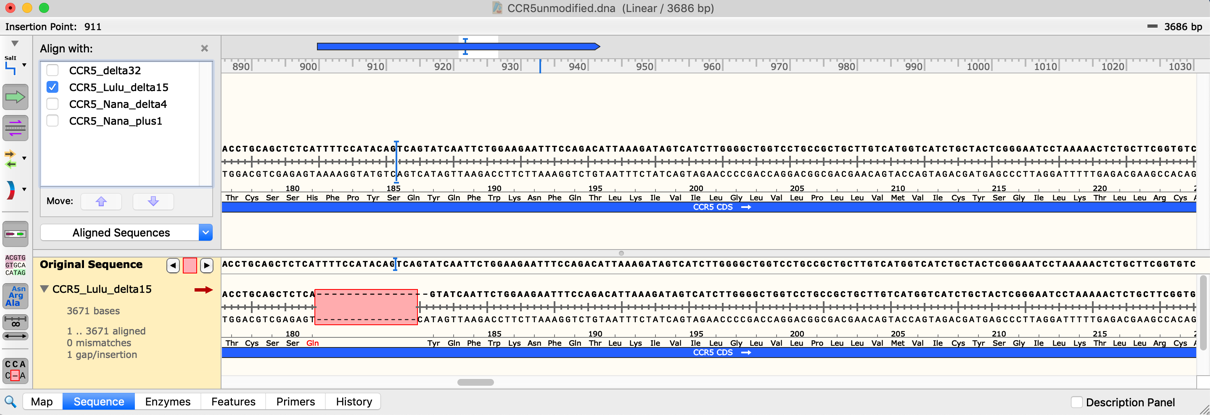


**Supplemental Figure 1:  Alignment of various *CCR5* alleles against unmodified *CCR5* allele**. Top window shows the unmodified CCR5 allele, and the bottom window shows the alignment with A) Δ

32 allele, B) Nana +1 allele, C) Nana Δ4 allele and D) Lulu Δ15 allele. Red boxes highlight mismatches, and red asterisk indicates stop codon that resulted from frameshift.

**Supplementary Figure 2:** Student pre- and post-survey self-reports on the A) effectiveness of CRISPR-cas9 technology in learning how authentic biology research is conducted and B) ability to apply tools learnt to experimental research and design.

**Supplementary Table 1: Bioethical Discussion Question on CRISPR germline editing**

|  | **Bioethics Discussion Questions** |
| --- | --- |
| **1** | What qualifies as a genetically modified organism?  How does somatic genetic editing differ from germline genetic editing? |
| **2** | Are there any fundamental differences between CRISPR-cas germ line editing in embryos vs gene therapy in children/adult patients?   - 1. How does gene editing in somatic cells vs germ cells differ?   2. Why do you think gene therapy is already an established part of biomedical research to treat disease, and CRISPR-cas9 based methods are currently approved and under clinical trials? |
| **3** | Discuss the types of human diseases that should utilize CRISPR-cas gene editing technology for gene therapy and your reasoning, |
| **4** | Is a regulating body that regulates the research actions of scientists internationally and nationally in the USA needed at this moment in time? If so, who should be in this regulating body? Should all countries have the same standards, or each country make their own? |
| **5** | Currently in the U.S., germline editing cannot be funded by federal grants. However, should there be oversight for experiments conducted by companies through private funding? |
| **6** | How should we proceed with other researchers who want to do germline editing/make genetically modified children to rid them of diseases-associated mutations? |
| **7** | Where do we draw the line between medical treatment versus enhancement? |

**Supplemental Table 2: *CCR5* CRISPR-cas gene editing-specific Discussion Questions**

|  | ***CCR5* CRISPR-cas case specific questions** |
| --- | --- |
| **1** | Was it medically necessary for Dr. He to inactivate CCR5 in these infants? |
| **2** | Should we be worried about the consequence of Dr. He creating novel alleles in the twin girls that were not the Δ32 allelic variant that confers natural HIV resistance? Given that these new alleles have unknown functions, should scientists test whether HIV immunity worked for these novel alleles? |
| **3** | Should there be consequences for Dr. He and his actions? If so, what consequences and by who? - It was reported that Dr. He was sentenced to 3 years in prison by Chinese government, has to pay $425,000 fine, and is banned for life from research in reproductive medicine [1]. |
| **4** | In response to Dr. He’s work, a Russian scientist has informed Nature Journal about his desire to create CCR5 genetically modified babies [2]. He claims to have already formed an agreement with a HIV center to recruited women infected with HIV to participate in his work. Should others be allowed to continue modifying CCR5 in embryos? |
| **5** | Currently human embryos used in research must be terminated after 14 days in countries like US and UK [3] . However, given the scientific outcry from the work of He and the bioethical implications of modifying human embryos, should there be a moratorium on research of human germline/embryo editing until further guidelines are established? |
| **6** | Recent studies show the CCR5 has important roles other than immune function. CCR5 plays a role in brain cognition, specifically that in mice that lack CCR5 memory improved [4]. In humans who are carriers for Δ32 CCR5 allele, individuals have a faster recovery from strokes [5]. Should Lulu and Nana be monitored by a specialized group of doctors and researchers for unintended consequences of their genetic edits since they have novel alleles that have not been studied before? |

**Supplementary Table 3: Rubric for Assessing Student Learning Objectives in Research Proposal**

| Student Learning Objectives | 0 - Does not meet | 1- Unacceptable | 2-Developing | 3- Acceptable | 4-Mastery |
| --- | --- | --- | --- | --- | --- |
| 1.Explain gene chosen for modification and the justification for gene and desired change in cell/organism of choice | Student did not complete this part of the assignment | No explanation of process or explanation contains significant errors | Limited explanation or significant gaps or errors in explanation and justification of desired change and cell/organism | Clear and sufficient explanation and justification of desired change and cell/organism, contains few errors or omissions | Sophisticated explanation and justification, including all relevant details |
| 2A.Determine target sequence/PAM Sequence | Student did not complete this part of the assignment | Student incorrectly identifies PAM sequence and target sequence (for example PAM and/target sequence is outside GFP gene sequence) | Student identifies PAM but chooses target site downstream to PAM instead of upstream; or identifies PAM sequence but chooses target sequence that includes 1 or 2 nts of PAM | Student identifies PAM and target site upstream to PAM but not at the most ideal position (should be 5’ of gene) | Student identifies PAM and target site upstream to PAM on correct strand and at correct location within gene |
| 2B. Determine the gRNA sequence utilized | Student did not complete this part of the assignment | Student incorrectly determines gRNA sequence | Student determines gRNA sequence but uses DNA nucleotides instead of RNA nucleotides | Student determines gRNA sequence but does not include 5’ and 3’ polarity of sequence | Student determines gRNA sequence accurately |
| 2C. Create repair template if HDR is utilized | Student did not complete this part of the assignment | Student incorrectly creates repair template | Student creates a repair template but with errors such as does not contain the desired change in RT | Student determines repair template sequence but with only a minor error (for example does not change the PAM sequence in RT), however the desired change MUST be included in RT | Student determines RT sequence accurately |
| 3. Describe how the effect of the gene modification will be measured if gene modification is successful, and expected results and challenges | Student did not complete this part of the assignment | No explanation of process or explanation contains significant errors | Limited explanation with minor errors | Clear and sufficient explanation, contains few errors or omissions | Sophisticated explanation including all relevant details of measurement and expected results |
| 4. Describe the bioethical implications of using CRISPR-cas9 system in your system | Student did not complete this part of the assignment | Description is inaccurate and incomplete | Limited description | Clear and sufficient description | Sophisticated description with multiple bioethical implications addressed from opposing sides |

**Supplementary Table 4: Rubric for Assessing Student Learning Objectives for Podcast Assignment**

| **CATEGORY** | **Exemplary** | **Proficient** | **Partially Proficient** | **Unsatisfactory** | **POINTS** |
| --- | --- | --- | --- | --- | --- |
| **Introduction & Conclusion** | **10 points** | **7 points** | **5 points** | **0 points** | **___/10** |
|  | Catchy, clever and concise introduction. Provides relevant information that audience can clearly comprehend. Establishes a clear purpose engaging the listener immediately. | Describes the topic. Engages the audience as the introduction proceeds. | Somewhat engaging. Provides a vague introduction that the audience can minimally comprehend. | Irrelevant or inappropriate introduction that does not engage listener. The purpose is vague and unclear. |  |
|  | Clearly describes who is speaking (host) and the names and background/expertise of interviewees | Describes most of the following: who is speaking, and names and background/expertise of interviewees. | Alludes to who is speaking, and names and background/expertise of interviewees. | Speaker is not identified.No introduction of names and background/expertise of interviewees. |  |
|  | Conclusion clearly and effectively summarizes key information for audience. | Conclusion summarizes information. | Conclusion vaguely summarizes key information | No conclusion is provided. |  |
| **Overall Content** | **20 points** | **14 points** | **10 points** | **0 points** | **___/20** |
|  | Creativity and original content enhance the purpose of the podcast in an innovative way. Accurate information and succinct concepts are presented. | Accurate information is provided succinctly. | Some information is inaccurate or long-winded. | Information is inaccurate. |  |
|  | Presented overall scientific content and background is highly appropriate for knowledge level of audience | Presented scientific content is mostly appropriate for audience | Presented scientific content is minimally appropriate for audience | Presented scientific content is not appropriate for audience |  |
|  | Vocabulary is highly appropriate for audience | Vocabulary is mostly appropriate. | Vocabulary is adequate. | Vocabulary is inappropriate for the audience. |  |
|  | Keeps focused on the topic. | Stays mainly on the topic. | Occasionally strays from the topic. | Does not stay on topic. |  |
| **Interview** | **10 points** | **7 points** | **5 points** | **0 points** | **___/10** |
|  | Host asks meaningful, stimulating and probing questions to draw relevant information from the interviewees | Host asks appropriate questions to interviewees | Host asks vague questions to interviewees | Only yes or no questions are used, or questions that are irreleevant. |  |
|  | Follow-up is used that draw further interesting information from the interviewee | Follow-up questions are used appropriately | Follow-up questions are occasionnaly irrelevant to the topic | No follow-up questions are asked |  |
|  | Interviewees convey specialized knowledge that is appropriate and engages audtience | Interviewees convey specialized knowledge that is appropriate and informative to the audience | Interviewees convey some minimally informative knowledge | No knowledge is provided by interviewees. |  |
|  | Host keeps interview progressing by creating smooth transitions between questions and answers | Host progesses interview by creating transitions | Host minimally progresses interivew | Host does not provide adequate transitions during interview |  |
| **Overall Delivery** | 10 points | 7 points | 5 point | 0 points | **___/10** |
|  | Well rehearsed, smooth delivery in a conversational style. Conversation between host and interviewees is very natural | Rehearsed, smooth delivery. Conversation between host and interviewes is mostly natural | Appears unrehearsed with uneven delivery. Conversation between host and interviewees is awkward and forced | Delivery is hesitant and chopyy, and sounds like presenter is reading. Conversation between host and interviewees in uncomfortable. |  |
|  | Highly effective enunciation, expression keep the audience listening. | Enunciation and expression are mostly effective. | Enunciation and expression are distracting. | Enunciation of spoken word is not clearly understandable or expression |  |
|  | Pace and rhythm of delivery is effective | Pace and rhythm of delivery is slightly too fast | Pace and rhythm of delivery is too fast | Pace of delivery is not effective at all; rhythm is distracting throughout the podcast. |  |
|  | Correct grammar is used throughout the podcast. | Correct grammar is mostly used during the podcast. | Occasionally incorrect grammar is used during the podcast. | Poor grammar is used throughout the podcast. |  |
| **TOTAL POINTS** |  |  |  |  | **___/50** |
| **Feedback and Comments** |  |  |  |  |  |
|  |  |  |  |  |  |

**Supplementary Table 5: Useful links**

| He Jiankui video announcement on YouTube | <https://www.youtube.com/watch?v=th0vnOmFltc> and the  <http://scienceofhiv.org/wp/?page_id=6> |
| --- | --- |
| Second International Summit on Human Genome Editing | <https://livestream.com/NASEM/events/8464254/videos/184103056>  Start at: 1:17:57 for the announcements) |
| CCR5 nucleotide sequence | <https://www.ncbi.nlm.nih.gov/nuccore/NM_000579> |
| CCR5 protein structure | (<https://www.rcsb.org/structure/4MBS>), |
| HIV animation | http://scienceofhiv.org/wp/?page_id=6. |
| SnapGene | <https://www.snapgene.com/> |
| University of Pennsylvania - T cell CRISPR therapy | <https://clinicaltrials.gov/ct2/show/NCT03399448?term=Crispr&rank=6>;  <https://www.pennmedicine.org/news/news-releases/2019/november/results-first-us-trial-crispr-edited-immune-cells-cancer-patients-safety-of-approach> |
| CRISPR therapeutics and Vertex  - beta globin gene to treat beta thalassemia | <http://ir.crisprtx.com/news-releases/news-release-details/crispr-therapeutics-and-vertex-announce-progress-clinical> |
| Editas - Leber Congenital Amaurosis type 10 (LCA10) | <https://clinicaltrials.gov/ct2/show/NCT03872479?term=Crispr&draw=3&rank=25>  <https://www.genengnews.com/news/editas-wins-fda-approval-for-ind-of-crispr-treatment-for-lca10/>  <https://nationalacademies.org/cs/groups/genesite/documents/webpage/gene_195245.pdf>  <https://www.editasmedicine.com/gene-editing-pipeline/> |

**References:**

1 A. Regalado He Jiankui faces three years in prison for CRISPR babies - MIT Technology Review n.d. Accessed on 12 February 2020. Available at: https://www.technologyreview.com/s/614997/he-jiankui-sentenced-to-three-years-in-prison-for-crispr-babies/.

2 D. Cyranoski Russian biologist plans more CRISPR-edited babies. (2019) *Nature*. **570**, 145–146.

3 J. B. Appleby, A. L. Bredenoord Should the 14‐day rule for embryo research become the 28‐day rule? (2018) *EMBO Mol. Med.* **10**,.

4 M. Zhou, S. Greenhill, S. Huang, T. K. Silva, Y. Sano, Y. Sano, et al. CCR5 is a suppressor for cortical plasticity and hippocampal learning and memory. (2016) *Elife*. **5**,.

5 M. T. Joy, E. Ben Assayag, D. Shabashov-Stone, S. Liraz-Zaltsman, J. Mazzitelli, M. Arenas, et al. CCR5 Is a Therapeutic Target for Recovery after Stroke and Traumatic Brain Injury. (2019) *Cell*. **176**, 1143-1157.e13.
